# *Plasmodium berghei* HMGB1 controls the host immune responses and splenic clearance by regulating the expression of *pir* genes

**DOI:** 10.1016/j.jbc.2024.107829

**Published:** 2024-09-27

**Authors:** Pradeep Mini Vaishalli, Rahul Das, Harveer Singh Cheema, Sourav Ghosh, Manjunatha Chandana, Aditya Anand, Krushna Chandra Murmu, Govindarajan Padmanaban, Balachandran Ravindran, Viswanathan Arun Nagaraj

**Affiliations:** 1 Infectious Disease Biology, Institute of Life Sciences, Bhubaneswar, Odisha, India; 2 Regional Centre for Biotechnology, Faridabad, Haryana, India; 3 Department of Botany, Meerut College, Meerut, Uttar Pradesh, India; 4 PharmaFace, Hyderabad, Telangana, India; 5 Department of Biochemistry, Indian Institute of Science, Bangalore, Karnataka, India

**Keywords:** malaria, parasite, HMGB1, recombinant protein expression, site-directed mutagenesis, gene knockout, inflammation, pathogenesis, host immune evasion, parasite clearance

## Abstract

High mobility group box (HMGB) proteins belong to the high mobility group (HMG) superfamily of non-histone nuclear proteins that are involved in chromatin remodeling, regulation of gene expression, and DNA repair. When extracellular, HMGBs serve as alarmins inducing inflammation, and this is attributed to the proinflammatory activity of box B. Here, we show that *Plasmodium* HMGB1 has key amino acid changes in box B resulting in the loss of TNF-α stimulatory activity. Site-directed mutagenesis of the critical amino acids in box B with respect to mouse HMGB1 renders recombinant *Plasmodium berghei* (*Pb*) HMGB1 capable of inducing TNF-α release. Targeted deletion of *Pb*HMGB1 and a detailed *in vivo* phenotyping show that *Pb*HMGB1 knockout (KO) parasites can undergo asexual stage development. Interestingly, Balb/c mice-infected with *Pb*HMGB1KO parasites display a protective phenotype with subsequent clearance of blood parasitemia and develop long-lasting protective immunity against the challenges performed with *Pb* wildtype parasites. The characterization of splenic responses shows prominent germinal centers leading to effective humoral responses and enhanced T follicular helper cells. There is also complete protection from experimental cerebral malaria in CBA/CaJ mice susceptible to cerebral pathogenesis with subsequent parasite clearance. Transcriptomic studies suggest the involvement of *Pb*HMGB1 in *pir* expression. Our findings highlight the gene regulatory function of parasite HMGB1 and its *in vivo* significance in modulating the host immune responses. Further, clearance of asexual stages in *Pb*HMGB1KO-infected mice underscores the important role of parasite HMGB1 in host immune evasion. These findings have implications in developing attenuated blood-stage vaccines for malaria.

Malaria caused by *Plasmodium* spp. is a serious health concern with tremendous economic impact responsible for 249 million infections and 608,000 deaths in 2022. With almost half of the world’s population at risk, children under 5 years of age and pregnant women are the most vulnerable (https://www.who.int/teams/global-malaria-programme/reports/world-malaria-report-2022). Human malaria is caused by five *Plasmodium* species - *falciparum*, *vivax*, *malariae*, *ovale*, and *knowlesi*, and more than 90% of malaria cases and deaths are due to *Plasmodium falciparum* (*Pf*) infections. *Plasmodium* has a complex life cycle that occurs in vertebrate (human) and invertebrate (mosquito) hosts (1). Transmitted by the female *Anopheline* mosquito, sporozoites introduced into the blood stream reach human liver and undergo exo-erythrocytic stage development in the hepatocytes. The exo-erythrocytic merozoites released from the hepatocytes invade red blood cells (RBCs) and undergo periodic asexual cycle giving rise to new merozoites that invade fresh RBCs until they are cleared by therapeutic interventions. A small fraction of merozoites gives rise to gametocyte formation in RBCs that are ingested by mosquitoes during the blood meal. These gametocytes form gametes, undergo fertilization and complete their sexual development to form sporozoites that invade salivary glands of the mosquitoes to initiate the transmission.

The periodic asexual cycle occurring in RBCs is responsible for the entire malaria pathogenesis and the asexual stage parasites deploy various strategies to evade host immune responses and splenic clearance. Besides the advantage of intracellular residing and the lack of MHC class I molecule expression in host RBCs, the malaria parasite - *P. falciparum* expresses multigene families like *var*, *rifin*, and *stevor* encoding variant surface antigens that are expressed mainly on the surface of infected RBCs (iRBCs) promoting cytoadherence and sequestration (2, 3, 4). By switching the expression of these highly polymorphic proteins through epigenetic mechanisms, the parasite camouflages them from the host immune responses (5, 6). It is also known that iRBCs are resistant to complement-mediated cell lysis and the knob-like features of *Pf* erythrocyte membrane protein 1 (EMP-1) encoded by *var* genes can prevent the adequate deposition of IgGs (7, 8). Further, the recognition of key pathogen-associated molecular patterns (PAMPs) of malaria parasite such as hemozoin, glycosylphosphatidylinositols (GPIs) and nucleic acids by pattern-recognition receptors and cytosolic sensors, often lead to pro-/anti-inflammatory and Th1/Th2 imbalances with aberrant effector and memory T cell responses (9, 10, 11). This is further augmented by the ability of phagocytosed iRBCs and hemozoin to cause premature apoptosis and thereby, preventing the innate immune responses of macrophages, monocytes and dendritic cells (11, 12). Also, there occurs an atypical memory B cell response impeding the antibody production and long-lived immunity (13). All these culminate in preventing the development of protective immunity against asexual stage parasites.

High mobility group box (HMGB) proteins are non-histone chromosome-binding nuclear proteins that contain at least one HMG-box domain and are expressed in almost all eukaryotes. Many eukaryotes contain a large number of HMGB proteins and most of them possess one or two HMG-boxes, although transcription factors like UBF1 contain up to six HMG-boxes (14). In addition to nuclear functions such as chromatin organization, transcriptional regulation, and DNA repair (15, 16, 17), HMGB proteins have evolved to perform cytosolic and extracellular functions. A typical example for the cytosolic function is the regulation of autophagy and mitophagy by binding of human/mouse HMGB1 to key autophagy proteins like Beclin1 and Atg5 through its active shuttling between nucleus and cytosol (18, 19). When present extracellular either through active secretion or passive release, HMGB1 serves as a cytokine or danger-associated molecular pattern (DAMP) by mediating the inflammation. Depending on the overall redox state and interaction with various receptors such as TLR4, RAGE, *and so on*, HMGB1 can induce proinflammatory responses (20, 21, 22).

The genome of the malaria parasite encodes four HMGB proteins—HMGB1, HMGB2, HMGB3, and HMGB4 that are conserved across the *Plasmodium* species (23). While HMGB1 and HMGB2 are of around 100 amino acids in length with one HMG-box, HMGB3 consists of over 2000 amino acids with two HMG-boxes and HMGB4 has around 250 amino acids with one HMG-box. Of the four HMGBs, HMGB1 and HMGB2 have been studied so far. *In vivo* studies carried out in mice infected with *Plasmodium berghei* (*Pb*; rodent parasite) HMGB2 knockout (KO) parasites have shown significant protection from experimental cerebral malaria (ECM), and pre-immunization with iRBCs could confer long-lasting sterile protection against homologous and heterologous *Pb* strains (24, 25). Another study carried out in the rodent parasite, *Plasmodium yoelii* (*Py*), has shown that the deletion of HMGB2 leads to a prominent reduction of oocyst formation in mosquitoes (26). While the former study suggests the proinflammatory function of HMGB2 being responsible for ECM, the latter highlights the gene regulatory function wherein, ∼30 genes with most of them expressed in the gametocyte stages are downregulated in HMGB2KO parasites. Similarly, deletion of HMGB2 in *Pf* has led to a significant reduction in oocyst formation with no prominent changes in asexual stage development (27). In case of HMGB1, a ChIP-seq study carried out with *Pf*HMGB1 wildtype (WT) and KO lines has shown that HMGB1 deletion disrupts centromere-/telomere-dependent nuclear architecture leading to a complete silencing of *var* expression (28). Given this background, we have sought to examine the proinflammatory nature of parasite HMGB1 and the *in vivo* phenotype of HMGB1 deletion using a lethal *Pb* ANKA strain. Our results suggest that the parasite HMGB1 lacks TNF-α stimulatory activity, and *Pb*HMGB1KO parasites can lead to a protective phenotype in the asexual stage infections with long-lasting immunity. The parasite HMGB1 can regulate the expression of *Plasmodium* interspersed repeat (*pir*) multigene families that are associated with host-immune evasion and malaria pathogenesis. These results indicate the important role played by HMGB1 in the asexual stages of malaria parasite.

## Results

### *Pb*HMGB1 lacks TNF-α stimulatory activity

**Figure 1:**
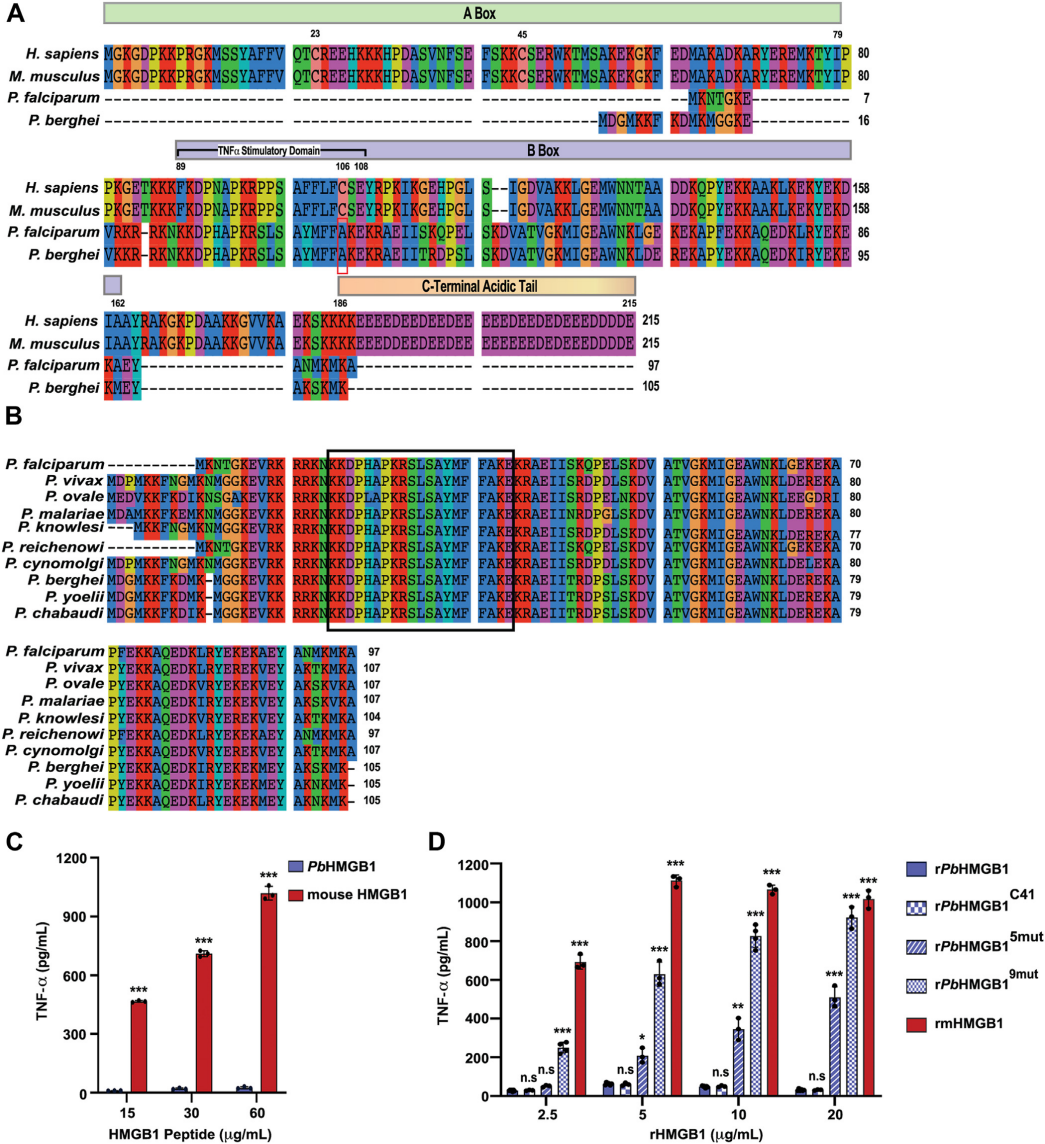
**Sequence comparison of parasite HMGB1 and the lack of TNF-α stimulatory activity.***A*, multiple protein sequence alignment of mouse, human, *Pf* and *Pb* HMGB1. The alignment shows the absence of A box and C-terminal acidic tail in *Plasmodia* HMGB1. The cysteine residues Cys23 and Cys45 present in the A box, and Cys106 present in the B box of mammalian (mouse and human) HMGB1 are highlighted with their respective numbers. The TNF-α stimulatory domain of mammalian HMGB1 (89–108 amino acids) and the corresponding sequence in *Plasmodia* HMGB1 are shown. The presence of indigenous Ala in the *Plasmodia* HMGB1 sequence corresponding to the TNF-α stimulatory domain of mammalian HMGB1 is highlighted in red box. *B*, multiple protein sequence alignment of *Plasmodia* HMGB1 infecting humans, rodents and primates. The 20 amino acid sequence in *Plasmodia* HMGB1 that corresponds to TNF-α stimulatory domain of mammalian HMGB1 is highlighted in a box. The respective sequence is conserved across the represented *Plasmodium* species with almost 100% identity. Multiple protein sequence alignments were carried out with SeaView Version 5.0.5 (https://doua.prabi.fr/software/seaview). *C*, treatment of murine macrophage-like RAW 264.7 cell line with the synthetic peptide of mouse HMGB1 representing TNF-α stimulatory domain of 20 amino acid length and the corresponding synthetic peptide of *Pb*HMGB1. After 12 h of treatment, ELISA was performed with the culture supernatants to estimate the levels of TNF-α secretion. The data (mean ± SD) represent three independent experiments (∗∗∗*p* < 0.001, unpaired *t* test; two-tailed). *D*, treatment of murine macrophage-like RAW 264.7 cell line with r*Pb*HMGB1, r*Pb*HMGB1^C41^, r*Pb*HMGB1^5mut^, r*Pb*HMGB1^9mut^ and rmHMGB1. After 12 h of treatment, ELISA was performed with the culture supernatants to estimate the levels of TNF-α secretion. The data (mean ± SD) represent at least three independent experiments. (n.s.- not significant, ∗*p* < 0.05, ∗∗*p* < 0.01, ∗∗∗*p* < 0.001; unpaired *t* test; two-tailed).

Multiple sequence alignment of *Plasmodia* (*Pf* and *Pb*) HMGB1 with mammalian (human and mouse) HMGB1 suggested ∼28% similarity and ∼20% identity. More importantly, *Plasmodia* HMGB1 lacks the A box and C-terminal acidic tail that are present in mammalian HMGB1 (Fig. 1*A*). The A box contains two critical cysteine residues (Cys23 and Cys45) whose redox status determines the chemoattractant and proinflammatory functions of mammalian HMGB1 (29). Further, the third critical cysteine residue (Cys106) present in the TNF-α stimulatory domain of B box in mammalian HMGB1 is also absent in *Plasmodia* HMGB1 (Fig. 1*A*). It has been shown that the mutation of Cys106 to Ala prevents the binding of mammalian HMGB1 to Toll-like receptor 4 (TLR4)/myeloid differentiation factor-2 (MD-2) complex and the subsequent release of TNF-α from macrophages (30). Interestingly, the sequence in *Plasmodia* HMGB1 corresponding to the TNF-α stimulatory domain of mammalian HMGB1 B box has an indigenous Ala in the place of Cys106 (Fig. 1*A*). Further, the characteristic features of parasite HMGB1 lacking A box, C-terminal acidic tail and Cys106 are conserved across the *Plasmodium* species infecting humans, primates, and rodents (Fig. 1*B*). All these prompted us to examine the TNF-α stimulatory activity of *Plasmodia* HMGB1.

It has already been shown that the synthetic peptide of 20 amino acids representing the TNF-α stimulatory domain of mammalian HMGB1 can lead to TLR-4-dependent activation of macrophages and TNF-α release (30, 31). Therefore, to begin with, we examined the TNF-α stimulatory activity of the *Pb*HMGB1 synthetic peptide of length 20 amino acids that corresponds to the TNF-α stimulatory domain of mammalian HMGB1 using a murine macrophage-like RAW 264.7 cell line. The respective 20 amino acid sequence of parasite HMGB1 has retained almost 100% identity within the *Plasmodia* species (Fig. 1*B*). While the synthetic peptide of mouse HMGB1 TNF-α stimulatory domain could lead to TNF-α release at a concentration of 15 μg/ml, the corresponding synthetic peptide of *Pb*HMGB1 did not cause prominent TNF-α release even at a concentration of 60 μg/ml (Fig. 1*C*). To rule out the possibility of additional sequence requirement for TNF-α stimulation, the full-length recombinant *Pb*HMGB1 (r*Pb*HMGB1) expressed in *E. coli* was used. The r*Pb*HMGB1 was purified using Ni^2+^-NTA resin, followed by S-Sepharose chromatography to remove additional protein impurities, and DNase I treatment and endotoxin removal to deplete DNA and lipopolysaccharide (LPS) contaminations, respectively (Fig. S1*A*). As observed for the synthetic peptide, r*Pb*HMGB1 failed to induce TNF-α release even at a high concentration of 20 μg/ml. In contrast, recombinant mouse HMGB1 (rmHMGB1) could induce a strong TNF-α release at a concentration of 2.5 to 5.0 μg/ml (Fig. 1*D*). Since Cys106 of mammalian HMGB1 plays an important role in TLR4-dependent TNF-α release, we performed site-directed mutagenesis and replaced the indigenous Ala41 of r*Pb*HMGB1 with Cys (r*Pb*HMGB1^C41^) (Fig. S1*B*). The r*Pb*HMGB1^C41^ was purified under identical conditions as described for r*Pb*HMGB1 (Fig. S1*A*). However, the replacement of Ala with Cys could not lead to any prominent increase in TNF-α release (Fig. 1*D*). This in turn suggested that *Pb*HMGB1 has additional modifications in the sequence corresponding to TNF-α stimulatory domain and a sequence comparison with mouse HMGB1 suggested another nine amino acid changes. We performed a first set of sequential site-directed mutagenesis with r*Pb*HMGB1^C41^ plasmid replacing Lys42, Lys24, Leu34, and Ser33 with Ser, Phe, Pro and Pro, respectively (Fig. S1*B*). The purified r*Pb*HMGB1 with five mutations (r*Pb*HMGB1^5mut^) including Cys41 showed ∼30% of TNF-α release with respect to mouse HMGB1 at a concentration of 5.0 μg/ml (Figs. S1, *A* and *B* and 1*D*). We further included four additional mutations replacing His28, Tyr37, Met38 and Phe39 with Asn, Phe, Phe, and Leu, respectively (Fig. S1, *A* and *B*). The resultant r*Pb*HMGB1 with nine mutations (r*Pb*HMGB1^9mut^) matched completely with TNF-α stimulatory domain of mouse HMGB1 and showed ∼60% of TNF-α release with respect to mouse HMGB1 at a concentration of 5.0 μg/ml. At concentrations of 10 to 20 μg/ml, the levels of TNF-α release were comparable with respect to mouse HMGB1 (Fig. 1*D*). These findings suggested that the *Plasmodia* HMGB1 lacks TNF-α stimulatory activity.

### *Pb*HMGB1 is undetectable in plasma

**Figure 2:**
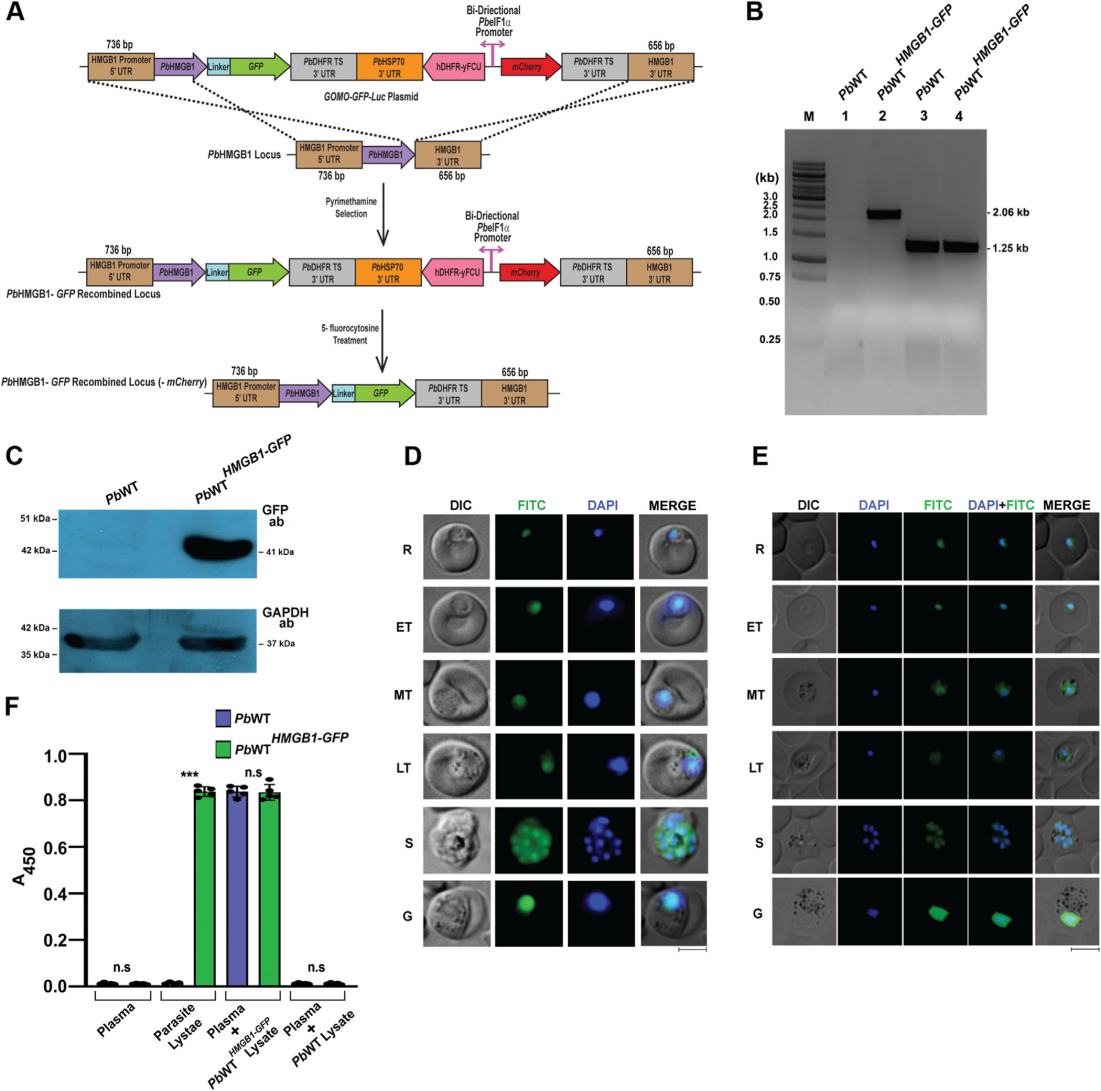
**Generation of *Pb*WT**^***HMGB1-GFP***^**parasites and examination of the extracellular presence of parasite HMGB1.***A*, schematic representation of double-crossover recombination strategy followed to generate *Pb*WT^*HMGB1-GFP*^ parasites. *B*, genomic DNA PCR confirmation for site-specific integration of HMGB1-GFP in *Pb*WT^HMGB1-GFP^ parasites. Lane M: 1 kb ladder. Lane 1 and 2: Integration-specific product of 2.06 kb amplified using forward primer upstream to the promoter sequence of *Pb*HMGB1 and GFP-specific reverse primer. Lane 3 and 4: *Pb*GAPDH control (1.25 kb). *C*, Western blot analysis of *Pb*HMGB1-GFP protein expression in *Pb*WT^*HMGB1-GFP*^ parasites. 100 μg of total protein was used. *D*, live imaging of *Pb*WT^*HMGB1-GFP*^ parasites showing the localization of endogenous *Pb*HMGB1-GFP. DAPI treatment was carried out to stain the nuclear DNA. Images were captured using 100× objective lens. Scale bar = 5 μm. *E*, indirect immunofluorescence analysis of *Pb*HMGB1-GFP localization in *Pb*WT^*HMGB1-GFP*^ parasites using GFP antibodies. DAPI treatment was carried out to stain the nuclear DNA. Images were captured using 100× objective lens. Scale bar = 5 μm. *F*, ELISA of plasma samples collected from *Pb*WT- and *Pb*WT^*HMGB1-GFP*^-infected mice using GFP antibodies. 100 μl plasma samples were used to coat the wells. For parasite lysate control, 50 μg of *Pb*WT and *Pb*WT^*HMGB1-GFP*^ lysates were used. For spiking, the respective plasma samples were spiked with 50 μg of *Pb*WT^*HMGB1-GFP*^ or *Pb*WT lysates. The data (mean ± SD) represent five different samples (n.s.- not significant, ∗∗∗*p* < 0.001; unpaired *t* test; two-tailed). ET, early trophozoite; G, gametocyte; LT, late trophozoite; MT, mid trophozoite; R, ring; S, schizont.

Our next interest was to examine whether *Pb*HMGB1 undergoes active secretion or passive release. For this, we generated *Pb*WT^*HMGB1-GFP*^ transgenic parasites through double-crossover recombination wherein, the C-terminus of endogenous *Pb*HMGB1 was tagged in-frame with a linker followed by green fluorescence protein (GFP) (Fig. 2*A*). The modification of *Pb*HMGB1 locus in *Pb*WT^*HMGB1-GFP*^ parasites was confirmed by genomic DNA PCR analysis (Fig. 2*B*). Western blot analysis carried out with the lysates of *Pb*WT^*HMGB1-GFP*^ parasites confirmed the expression of a 41 kDa *Pb*HMGB1-GFP fusion protein (Fig. 2*C*). Live imaging studies performed with *Pb*WT^*HMGB1-GFP*^ transgenic parasites showed the co-localization of GFP fluorescence with the DNA staining of 4′,6-diamidino-2-phenylindole (DAPI) suggesting the nuclear localization of *Pb*HMGB1 (Fig. 2*D*). These results were further confirmed by indirect immunofluorescence analysis performed with GFP antibodies (Fig. 2*E*). To examine the extracellular presence of *Pb*HMGB1, plasma samples were collected from mice infected with *Pb*WT^*HMGB1-GFP*^ parasites showing ∼10% blood parasitemia (∼10^9^ parasites/ml of blood) and ELISA was carried out with GFP antibodies. The GFP antibodies could detect *Pb*HMGB1-GFP at as low as 10^4^ *Pb*WT^*HMGB1-GFP*^ parasites (Fig. S2*A*). For control, plasma samples of *Pb*WT-infected mice were used. The results obtained suggested that *Pb*HMGB1-GFP could not be detected in the plasma samples of *Pb*WT^*HMGB1-GFP*^-infected mice (Fig. 2*F*). There was only a background signal as observed for *Pb*WT-infected mice plasma. However, GFP antibodies could react with the endogenous *Pb*HMGB1-GFP present in the *Pb*WT^*HMGB1-GFP*^ parasite lysates. To further ensure these findings, the respective plasma samples were independently spiked with the lysates of *Pb*WT^*HMGB1-GFP*^ and *Pb*WT parasites. As expected, GFP antibodies could readily detect the plasma samples spiked with *Pb*HMGB1-GFP lysates, but not with *Pb*WT lysates (Fig. 2*F*). All these results obtained with *Pb*WT^*HMGB1-GFP*^ parasites suggested that *Pb*HMGB1 remains undetectable in the plasma samples of infected mice and it is neither actively secreted nor passively released.

### *Pb*HMGB1 deletion leads to the clearance of asexual stages

**Figure 3:**
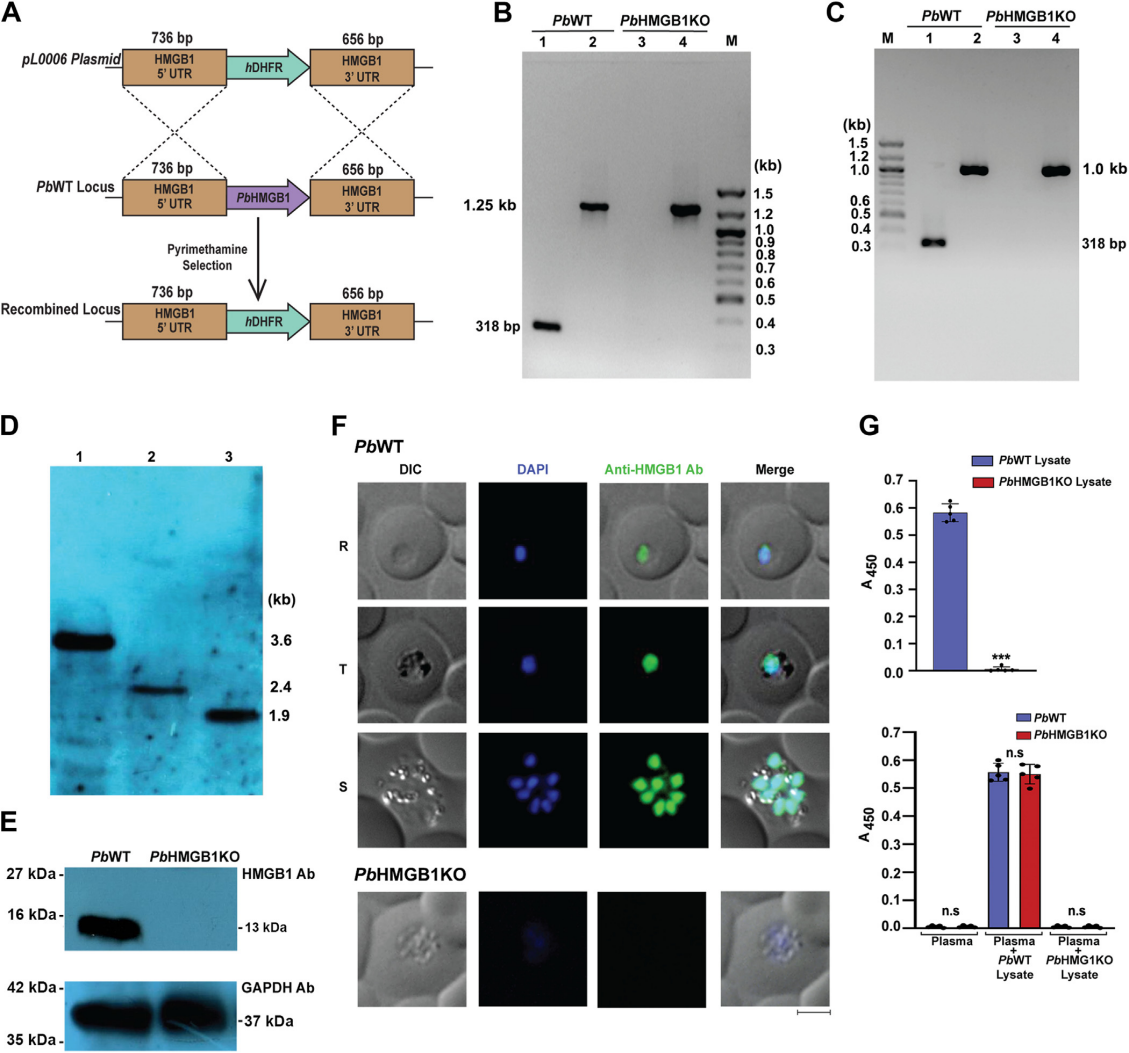
**Generation of *Pb*HMGB1KO parasites.***A*, double cross-over recombination strategy followed to generate *Pb*HMGB1KO parasites. *B,* genomic DNA PCR confirmation of HMGB1 deletion in *Pb*HMGB1KO parasites. Lane 1 and 3: PCR amplification of *Pb*HMGB1 (318 bp). Lane 2 and 4: *Pb*GAPDH control (1.25 kb). Lane M: 100 bp ladder. *C*, RT-PCR confirmation of HMGB1 deletion in *Pb*HMGB1KO parasites. Lane 1 and 3: PCR amplification of HMGB1 (318 bp). Lane 2 and 4: *Pb*GAPDH control (1.0 kb). Lane M: 100 bp ladder. *D*, southern blot confirmation of site-specific integration in *Pb*HMGB1KO parasites. Lane 1: Recombinant plasmid used for transfection as a control to rule out the presence of episomes. Lane 2 and 3: Genomic DNA isolated from *Pb*WT and *Pb*HMGB1KO parasites, respectively. Genomic DNA and plasmid samples were digested with *SphI* and *HindIII* and hybridized with 3′UTR-specific probe of *Pb*HMGB1. *Pb*WT and *Pb*HMGB1KO genomic DNA showed the hybridized fragments of size 2.4 kb and 1.9 kb, respectively. For recombinant plasmid, the fragment size was 3.6 kb. *E*, Western blot confirmation of HMGB1 deletion in *Pb*HMGB1KO parasites using *Pb*HMGB1 polyclonal antibodies. 150 μg of total protein was used for SDS-PAGE. *F*, immunofluorescence analysis of HMGB1 localization in *Pb*WT parasites and HMGB1 deletion in *Pb*HMGB1KO parasites. Scale bar = 5 μM. *G*, ELISA analysis of plasma samples collected from *Pb*WT- and *Pb*HMGB1KO-infected mice using polyclonal *Pb*HMGB1 antibodies. 100 μl plasma samples were used to coat the wells. For parasite lysates, 50 μg of *Pb*WT and *Pb*HMGB1KO lysates were used. The data (mean ± SD) represent five different samples (n.s.- not significant, ∗∗∗*p* < 0.001; unpaired *t* test; two-tailed).

**Figure 4:**
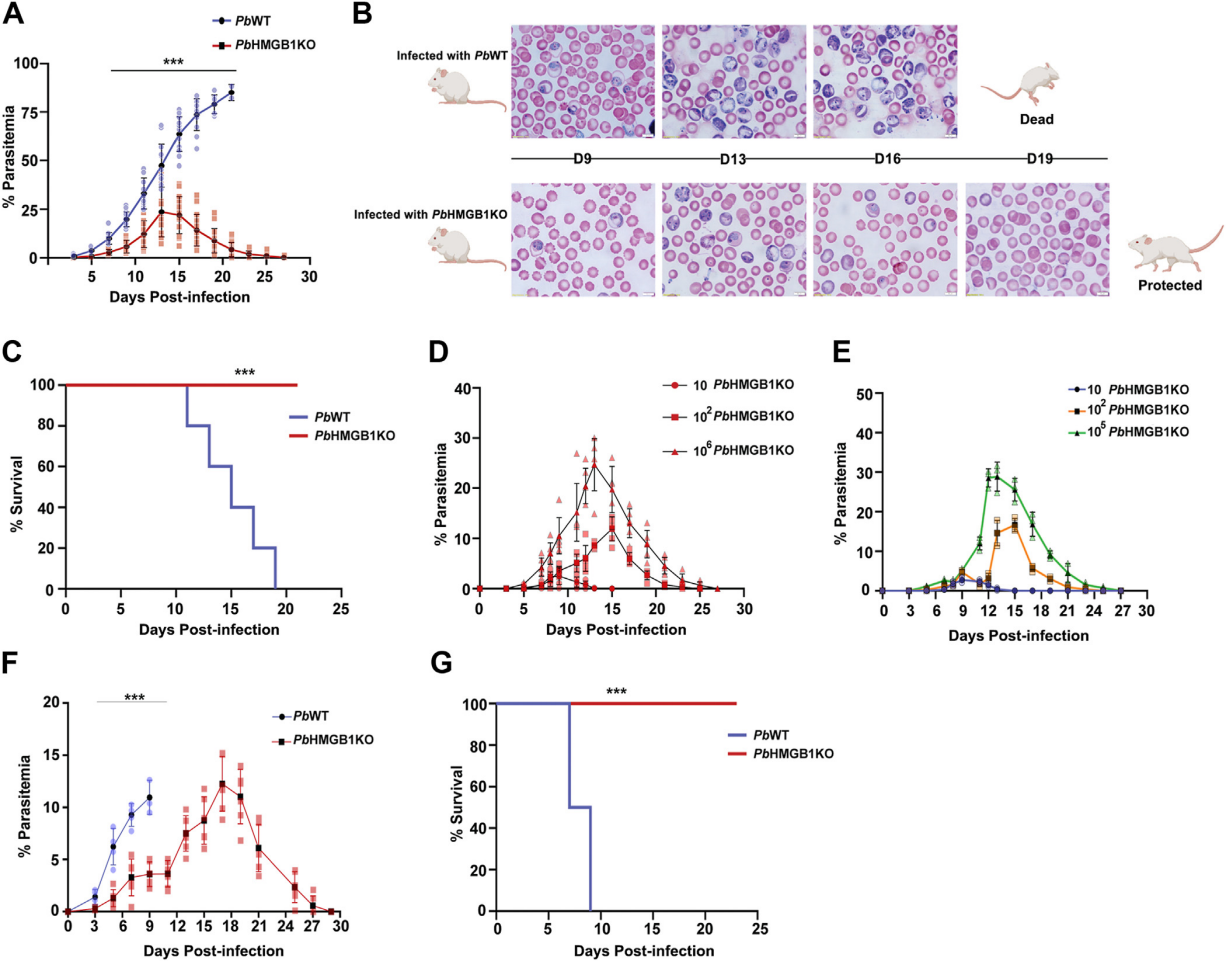
**Characterization of a sexual phenotype of *Pb*HMGB1KO parasites.***A*, growth analysis of *Pb*WT (n = 13) and *Pb*HMGB1KO (n = 16) in Balb/c mice. 10^5^ parasites were used for infection. The data (mean ± SD) represent three different batches (∗∗∗*p* < 0.001; Two-way ANOVA; Tukey test). *B*, Geimsa-stained images of blood smears prepared from tail vein blood of *Pb*WT and *Pb*HMGB1KO parasite-infected mice. Scale bar = 10 μM. *C*, Survival analysis of Balb/c mice infected with *Pb*WT (n = 5) and *Pb*HMGB1KO (n = 11) parasites (∗∗∗*p* < 0.001; log-rank (Mantel-Cox) test). *D*, clearance of blood parasitemia in Balb/c mice (n = 6) infected with 10, 10^2^ or 10^6^ parasitized RBCs through intravenous route. The data (mean ± SD) represent three different batches. (∗∗∗*p* < 0.001; Two-way ANOVA; Tukey test). *E*, growth analysis of *Pb*HMGB1KO parasites collected during protective phase. The naïve Balb/c mice (n = 3) were infected with 10, 10^2^ or 10^5^ parasitized RBCs through intravenous route. *F*, growth analysis of *Pb*WT (n = 4) and *Pb*HMGB1KO (n = 6) in CBA/CaJ mice. 10^5^ parasites were used for infection. The data (mean ± SD) represent three different batches (∗∗∗*p* < 0.001; Two-way ANOVA; Tukey test). *G*, survival analysis of CBA/CaJ mice (n = 12) infected with *Pb*WT and *Pb*HMGB1KO parasites (∗∗∗*p* < 0.001 log-rank (Mantel-Cox) test).

To understand the significance of HMGB1 in the life cycle of *Pb*, targeted deletion was carried out through double-crossover recombination (Fig. 3*A*). The successful deletion of *Pb*HMGB1 was verified by genomic DNA PCR, RT-PCR and Southern analyses (Fig. 3, *B*–*D*). We raised polyclonal antibodies against r*Pb*HMGB1 and Western analysis carried out with the lysates of *Pb*WT and *Pb*HMGB1KO parasites showed the presence of a 13 kDa *Pb*HMGB1 in *Pb*WT, but not in *Pb*HMGB1KO parasites (Fig. 3*E*). This was also true for immunofluorescence analysis wherein, nuclear localization of *Pb*HMGB1 could only be detected in *Pb*WT parasites, but not in the *Pb*HMGB1KO parasites (Fig. 3*F*). Having confirmed the specificity, we further utilized *Pb*HMGB1 polyclonal antibodies to verify the ELISA results obtained for the absence of extracellular HMGB1 in *Pb*WT^*HMGB1-GFP*^-infected mice with GFP antibodies. The *Pb*HMGB1 polyclonal antibodies could detect at least 0.1 to 1.0 ng of r*Pb*HMGB1, and also the native *Pb*HMGB1 at as low as 10^4^ *Pb*WT parasites (Fig. S2, *B* and *C*). Once again, *Pb*HMGB1 antibodies could only detect *Pb*HMGB1 in *Pb*WT parasite lysates, but not in the plasma samples of the *Pb*WT-infected mice. As expected, no reactivity could be observed for *Pb*HMGB1KO parasite lysates and plasma samples of *Pb*HMGB1KO-infected mice (Fig. 3*G*). Our next interest was to examine the asexual phenotype of *Pb*HMGB1KO parasites. Growth analysis performed for the asexual stages in Balb/c mice infected with 10^5^ *Pb*HMGB1KO parasitized RBCs through intraperitoneal route suggested a delay of ∼3 days in comparison with *Pb*WT parasite growth. Importantly, mice infected with *Pb*HMGB1KO parasites displayed a subsequent clearance when the blood parasitemia reached around 15 to 30% (Fig. 4, *A* and *B*). While all the mice infected with *Pb*WT parasites succumbed to anemia within day 19 post-infection, *Pb*HMGB1KO-infected mice were completely protected (Fig. 4*C*). To rule out any reappearance of asexual stage parasites in the blood due to recrudescence, the protected mice were monitored over a period of 6 months by examining the presence of parasites in tail vein blood smears prepared at an interval of 5 days. However, no parasites could be detected suggesting the complete clearance of blood parasitemia and the absence of recrudescence.

The clearance of *Pb*HMGB1KO parasites and the protective phenotype of *Pb*HMGB1KO-infected mice were also reproducible with the inoculum of 10, 10^2^, or 10^6^ parasitized RBCs through the intravenous route (Fig. 4*D*). To rule out the subsequent clearance of *Pb*HMGB1KO asexual stage infections occurring due to any growth defect in the parasite *per se*, *Pb*HMGB1KO parasites were collected during the protective phase when the blood parasitemia fell to 0.1%. When such parasites were injected into naive Balb/c mice, they could successfully establish the blood stage infections leading to an increase in the blood parasitemia followed by subsequent clearance (Fig. 4*E*). We next examined the protective phenotype of *Pb*HMGB1KO parasites in CBA/CaJ mice - an *in vivo* mouse model for ECM, by injecting 10^5^ *Pb*HMGB1KO-parasitized RBCs through the intraperitoneal route. As observed for Balb/c mice, *Pb*HMGB1KO parasites displayed a growth delay in CBA/CaJ mice (Fig. 4*F*). While all the *Pb*WT-infected CBA/CaJ mice died within 9 days post-infection with ∼80% of them showing the typical symptoms of ECM, none of the *Pb*HMGB1KO-infected mice showed ECM. There was also a complete protection from mortality due to ECM and/or anemia with subsequent clearance of blood parasitemia (Fig. 4*G*). All these findings suggested the important role played by parasite HMGB1 in sustaining the *in vivo* asexual stage infections.

### Splenic clearance of *Pb*HMGB1KO parasites

**Figure 5:**
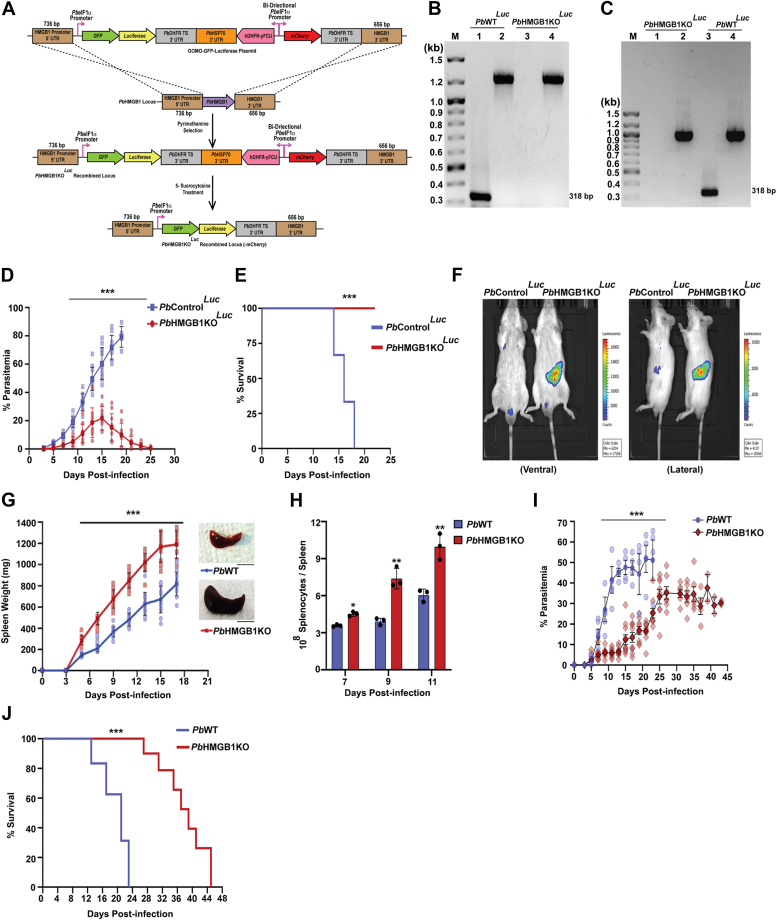
***In vivo* bioluminescence studies with *Pb*HMGB1KO**^***Luc***^**parasites and the effect of splenectomy on the clearance of *Pb*HMGB1KO parasites.***A*, double cross-over recombination strategy followed to generate *Pb*HMGB1KO^*Luc*^ parasites. *B*, genomic DNA PCR confirmation of HMGB1 deletion in *Pb*HMGB1KO^*Luc*^ parasites. Lane M: 100 bp ladder. Lane 1 and 3: PCR amplification of HMGB1 (318 bp). Lane 2 and 4: GAPDH control (1.25 kb). *C*, RT-PCR confirmation of HMGB1 deletion in *Pb*HMGB1KO^*Luc*^ parasites. Lane M: 100 bp ladder. Lane 1 and 3: PCR amplification of HMGB1 (318 bp). Lane 2 and 4: *Pb*GAPDH control (1.0 kb). Lane M: 100 bp ladder. *D*, growth analysis of *Pb*Control^*Luc*^ (n = 10) and *Pb*HMGB1KO^*Luc*^ (n = 10) in Balb/c mice. 10^5^ parasites were used for infection. The data (mean ± SD) represent three different batches (∗∗∗*p* < 0.001; Two-way ANOVA; Tukey test). *E*, survival analysis of Balb/c mice infected with *Pb*Control^*Luc*^ (n = 6) and *Pb*HMGB1KO^*Luc*^ (n = 6) parasites (∗∗∗*p* < 0.001, log rank (Mantel-Cox) test). *F*, *in vivo* bioluminescence imaging of Balb/c mice infected with *Pb*Control^*Luc*^ and *Pb*HMGB1KO^*Luc*^ parasites. 10^5^ parasites were used for infection. *G*, Spleen weight of Balb/c mice infected with *Pb*WT (n = 14) and *Pb*HMGB1KO (n = 16). The data (mean ± SD) represent three different batches (∗∗∗*p* < 0.001; Two way ANOVA; Tukey test). Scale bar = 1 cm. *H*, total number of splenocytes from *Pb*WT- and *Pb*HMGB1KO-infceted mouse spleen. The data (mean ± SD) represent three mice (∗*p* < 0.05, ∗∗*p* < 0.01; unpaired *t* test; two-tailed). *I*, growth analysis of *Pb*WT (n = 5) and *Pb*HMGB1KO (n = 10) parasites in splenectomized Balb/c mice. 10^5^ parasites were used for infection. The data represent mean ± SD; ∗∗∗*p* < 0.001; Two-way ANOVA; Tukey test). *J*, survival analysis of splenectomized Balb/c mice infected with *Pb*WT (n = 4) and *Pb*HMGB1KO (n = 7) parasites; (∗∗∗*p* < 0.001; log-rank (Mantel-Cox) test].

We sought to verify the protective phenotype of *Pb*HMGB1KO parasites with an independent transgenic deletion line wherein, *Pb*HMGB1 was replaced with GFP-luciferase (*Pb*HMGB1KO^*Luc*^) through double-crossover recombination (Fig. 5*A*). The targeted deletion was confirmed by genomic DNA (Fig. 5*B*) and RT-PCR (Fig. 5*C*) analyses. As observed for *Pb*HMGB1KO parasites, mice infected with *Pb*HMGB1KO^*Luc*^ parasites showed protective phenotype in asexual stage infections. The blood parasitemia reached to a maximum of ∼25%, followed by the clearance with complete protection from mortality due to anemia (Fig. 5, *D* and *E*). Since *Pb*HMGB1KO^*Luc*^ parasites expressed luciferase, they offered the advantage of performing bioluminescence studies to track the parasites *in vivo*. Bioluminescence imaging carried out on day 7 post-infection showed prominent signal in the spleen of mice infected with *Pb*HMGB1KO^*Luc*^ parasites (Fig. 5*F*). This was observed despite the blood parasitemia of *Pb*HMGB1KO^*Luc*^-infected mice being less when compared with *Pb*WT-infected mice, suggesting an important role played by spleen in the clearance of *Pb*HMGB1KO^*Luc*^ parasites. In agreement, mice infected with *Pb*HMGB1KO parasites displayed splenomegaly with ∼2 times increase in the spleen weight with respect to *Pb*WT-infected mice (Fig. 5*G*). The increase in spleen weight of *Pb*HMGB1KO-infected mice was reflected in the total number of splenocytes (Fig. 5*H*). These findings prompted us to examine the asexual growth of *Pb*HMGB1KO parasites in splenectomized mice. Interestingly, the splenectomized mice could not clear the asexual stages of *Pb*HMGB1KO parasites and they died due to anemia (Fig. 5, *I* and *J*). However, there was a significant delay in mortality in comparison with *Pb*WT-infected mice and this could be due to the relatively slow growth of *Pb*HMGB1KO parasites (Fig. 5*J*). These results suggested that splenic clearance is responsible for the protective phenotype of mice infected with *Pb*HMGB1KO parasites.

### Genetic complementation in *Pb*HMGB1KO parasites restores the lethal phenotype

**Figure 6:**
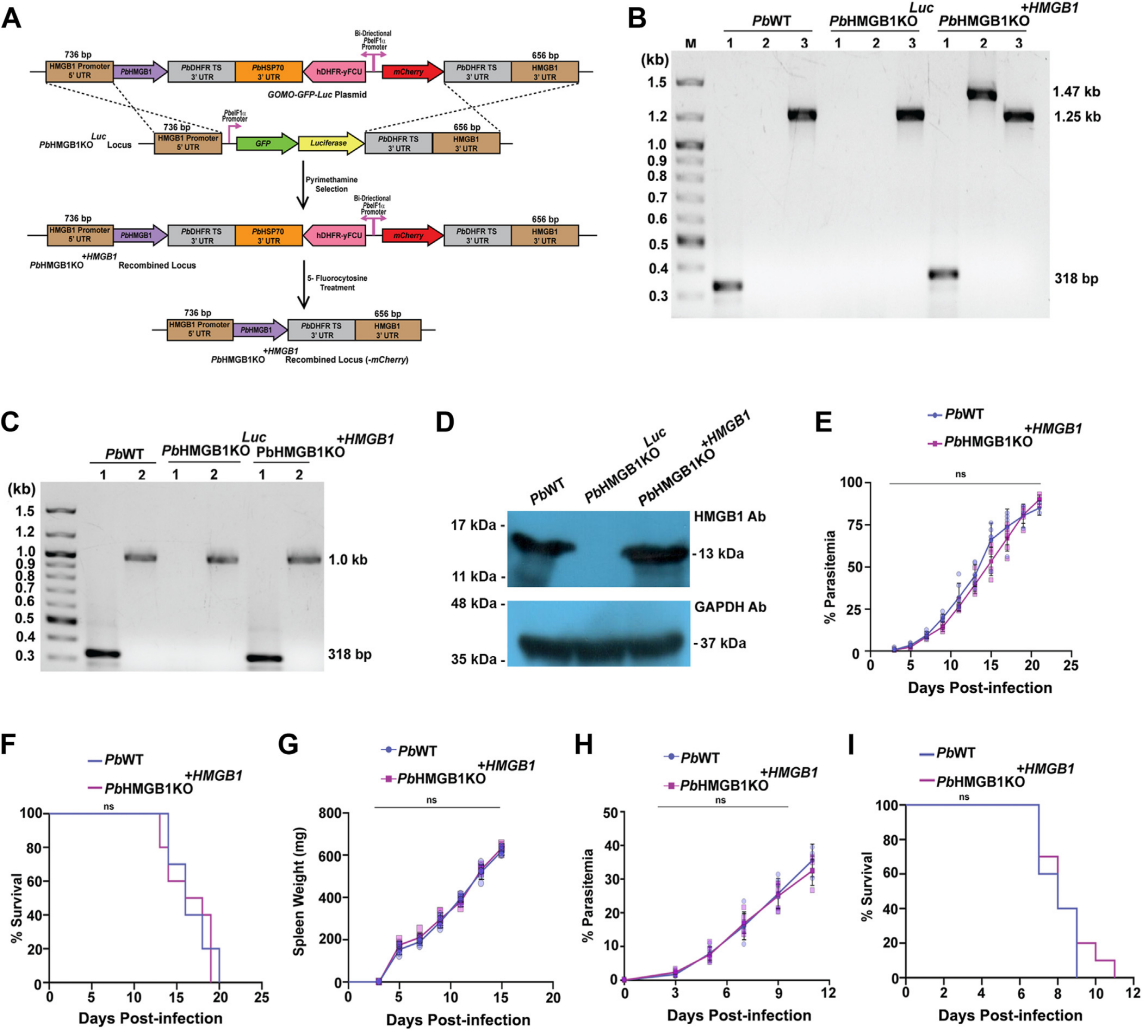
**Genetic complementation of *Pb*HMGB1 in *Pb*HMGB1KO**^***Luc***^**parasites.***A*, double cross-over recombination strategy followed to generate *Pb*HMGB1KO^*+HMGB1*^ parasites. The arrows denote the promoters present in the plasmid. The expression of *Pb*HMGB1 was restored at its endogenous locus with its own promoter. *B*, genomic DNA PCR confirmation for the genetic complementation of *Pb*HMGB1 and its site-specific integration in *Pb*HMGB1KO^*Luc*^ parasites. *C*, RT-PCR confirmation showing the expression of *Pb*HMGB1 RNA in *Pb*HMGB1KO^*+HMGB1*^ parasites. *D*, Western analysis showing the expression of *Pb*HMGB1 in *Pb*HMGB1KO^*+HMGB1*^ parasites. *E*, Growth analysis of *Pb*HMGB1KO^*+HMGB1*^ parasites in Balb/c mice (n = 6). 10^5^ parasites were used for infection. For control, *Pb*WT-infected mice (n = 6) were used. The data (mean ± SD) represent two different batches (n.s., not significant; Two-way ANOVA; Tukey test). *F*, survival analysis of Balb/c mice infected with *Pb*HMGB1KO^*+HMGB1*^ parasites. The data represent ten mice (n.s., not significant; log-rank (Mantel-Cox) test). *G*, Spleen weight of Balb/c mice infected with *Pb*WT (n = 6) and *Pb*HMGB1KO^*+HMGB1*^ parasites (n = 6). *H*, growth analysis of *Pb*HMGB1KO^*+HMGB1*^ parasites (n = 6) in CBA/CaJ mice. 10^5^ parasites were used for infection. For control, *Pb*WT-infected mice (n = 6) were used. The data (mean ± SD) represent two different batches (n.s., not significant; Two-way ANOVA; Tukey test). *I*, survival analysis of CBA/CaJ mice infected with *Pb*WT and *Pb*HMGB1KO parasites. The data represent ten mice (n.s., not significant; log-rank (Mantel-Cox) test).

To further ensure the findings on the protective phenotype of *Pb*HMGB1KO parasites, *Pb*HMGB1KO^*Luc*^-infected mice were treated with 5-fluorocytosine to remove the hDHFR-yFCU selection cassette. The transgenic parasites lacking pyrimethamine selection marker were then subjected to transfection for reintroducing *Pb*HMGB1 into its endogenous locus (Fig. 6*A*). The genetic complementation of *Pb*HMGB1 in the KO parasites (*Pb*HMGB1KO^*+HMGB1*^) and its site-specific integration were confirmed by genomic DNA and RT-PCR analyses (Fig. 6, *B* and *C*). Western analysis with the lysates of *Pb*HMGB1KO^*+HMGB1*^parasites suggested the restoration of *Pb*HMGB1 protein expression to the levels comparable with the *Pb*WT parasites (Fig. 6*D*). As expected, *Pb*HMGB1KO^*+HMGB1*^parasites could regain the lethal phenotype of *Pb*WT parasites and all the Balb/c mice infected with *Pb*HMGB1KO^*+HMGB1*^parasites died of anemia within day 17 post-infection (Fig. 6, *E* and *F*). The spleen weight of mice infected with *Pb*HMGB1KO^*+HMGB1*^parasites was similar to that of *Pb*WT-infected mice (Fig. 6*G*). There was also restoration of ECM phenotype and mortality in CBA/CaJ mice infected with *Pb*HMGB1KO^*+HMGB1*^parasites (Fig. 6, *H* and *I*). The findings from genetic complementation studies further confirmed that the protective phenotype observed in *Pb*HMGB1KO-infected mice is indeed due to the deletion of *Pb*HMGB1.

### Splenic architecture and germinal center responses in *Pb*HMGB1KO-infected mice

**Figure 7:**
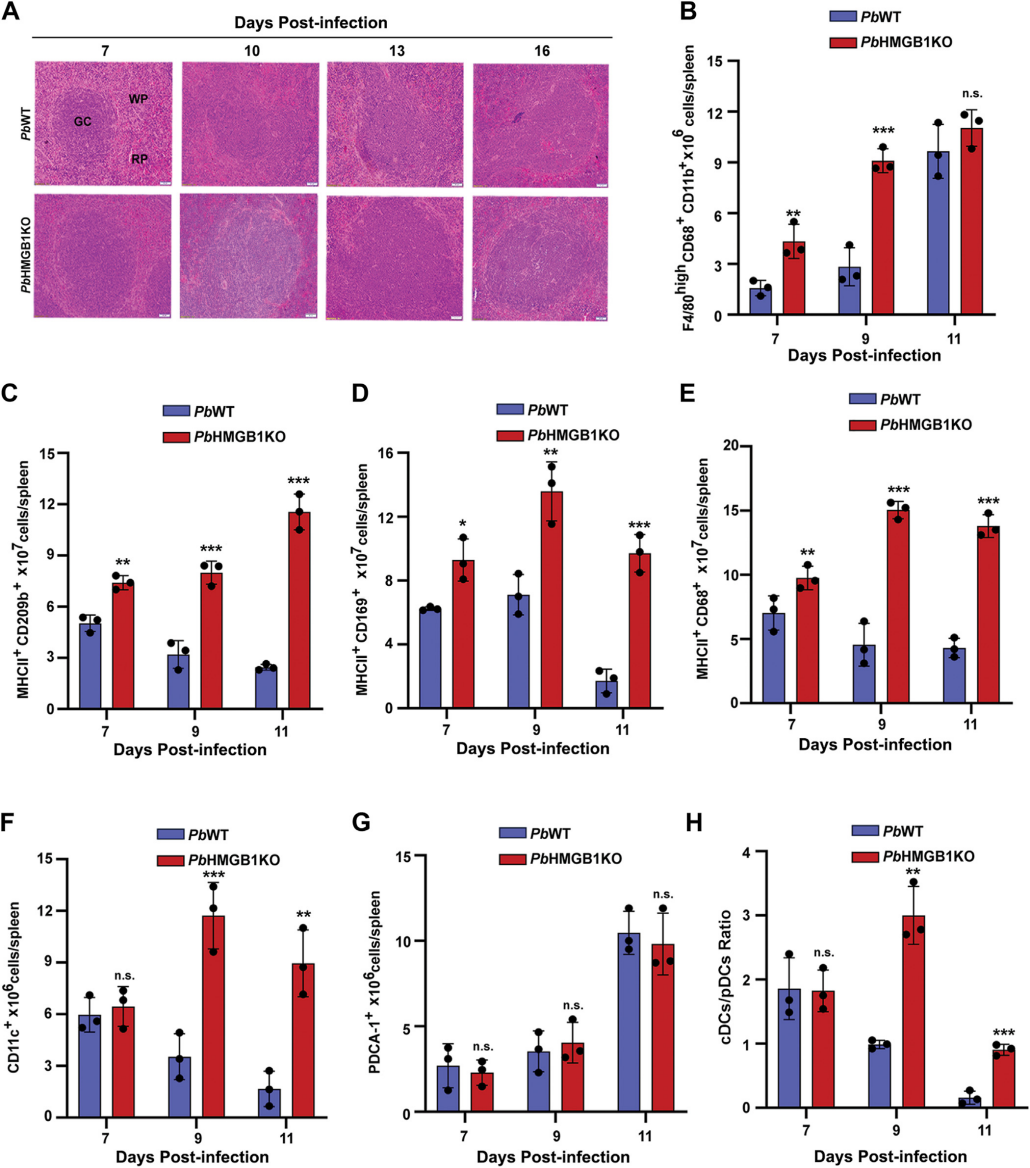
**Assessment of splenic architecture, macrophages and dendritic cells in *Pb*HMGB1KO-infected mice.***A*, H&E stained spleen sections of mice infected with *Pb*WT and *Pb*HMGB1KO parasites. The spleen samples were collected on the respective days post-infection. Images were captured using 10× objective. Scale bar = 50 μm. n = 3 independent experiments. *B–G*, Flow cytometry analyses of F4/80^high^ CD68^+^ CD11b^+^ red pulp macrophages (B), MHCII^+^ CD209b^+^ marginal zone macrophages (*C*), MHCII^+^ CD169^+^ marginal metallophilic macrophages (*D*), MHCII^+^ CD68^+^ tingible body macrophages (*E*), CD11c^+^ cDCs (*F*) and PDCA-1^+^ pDCs (*G*). The spleen samples of *Pb*WT- and *Pb*HMGB1KO-infected mice were collected and the total splenocytes were prepared to carry out the isolation and staining of macrophages and dendritic cells. The flow cytometry data (mean ± SD) represents three different mice for the respective days (∗*p* < 0.05, ∗∗*p* < 0.01, ∗∗∗*p* < 0.001, n.s., not significant; unpaired *t* test; two-tailed). *H*, cDCs/pDCs ratio in the spleen samples of *Pb*WT- and *Pb*HMGB1KO-infected mice. The data represent the mice used for flow cytometry analysis of cDCs and pDCs (n.s., not significant, ∗∗*p* < 0.01, ∗∗∗*p* < 0.001; unpaired *t* test; two-tailed). GC, germinal centers; RP, red pulp; WP, white pulp.

The dissolution of splenic architecture occurs in malaria infections (32, 33). Hematoxylin and eosin (H&E)-staining of the spleen sections suggested that the splenic architecture of *Pb*HMGB1KO-infected mice differed significantly from *Pb*WT-infected mice. While the demarcations of red pulp and white pulp compartments were comparable between *Pb*WT- and *Pb*HMGB1KO-infected mice on day 7 post-infection, there was a progressive disruption in the splenic architecture of *Pb*WT-infected mice on subsequent days. In contrast, *Pb*HMGB1KO-infected mice displayed a well-preserved splenic architecture throughout the infection. The presence of intact and well-demarcated red and white pulp regions in the spleen sections of *Pb*HMGB1KO-infected mice could be observed even on day 16 post-infection corresponding to the clearance phase (Fig. 7*A*). Flow cytometry analysis showed a significant increase in the red pulp macrophages (F4/80^high^ CD68^+^ CD11b^+^) of *Pb*HMGB1KO-infected mouse spleen on Days 7 and 9 post-infection (Figs. 7*B* and S3). There was also a prominent increase in the marginal zone and white pulp macrophages such as marginal zone macrophages (MHCII^+^ CD209b^+^), marginal metallophilic macrophages (MHCII^+^ CD169^+^), and tingible body macrophages (MHCII^+^ CD68^+^) (Figs. 7, *C*–*E* and S4) between day 7 and 11 post-infection. Similarly, there was a significant increase in the CD11c^+^ conventional dendritic cells (cDCs) of *Pb*HMGB1KO-infected mouse spleen on Days 9 and 11 (Figs. 7*F* and S5). However, the levels of PDCA-1^+^ plasmacytoid dendritic cells (pDCs) in the spleen remained comparable between *Pb*WT- and *Pb*HMGB1KO-infected mice (Figs. 7*G* and S5). This was also reflected in the ratio of cDCs to pDCs in the spleen of *Pb*HMGB1KO-infected mouse (Fig. 7*H*).

**Figure 8:**
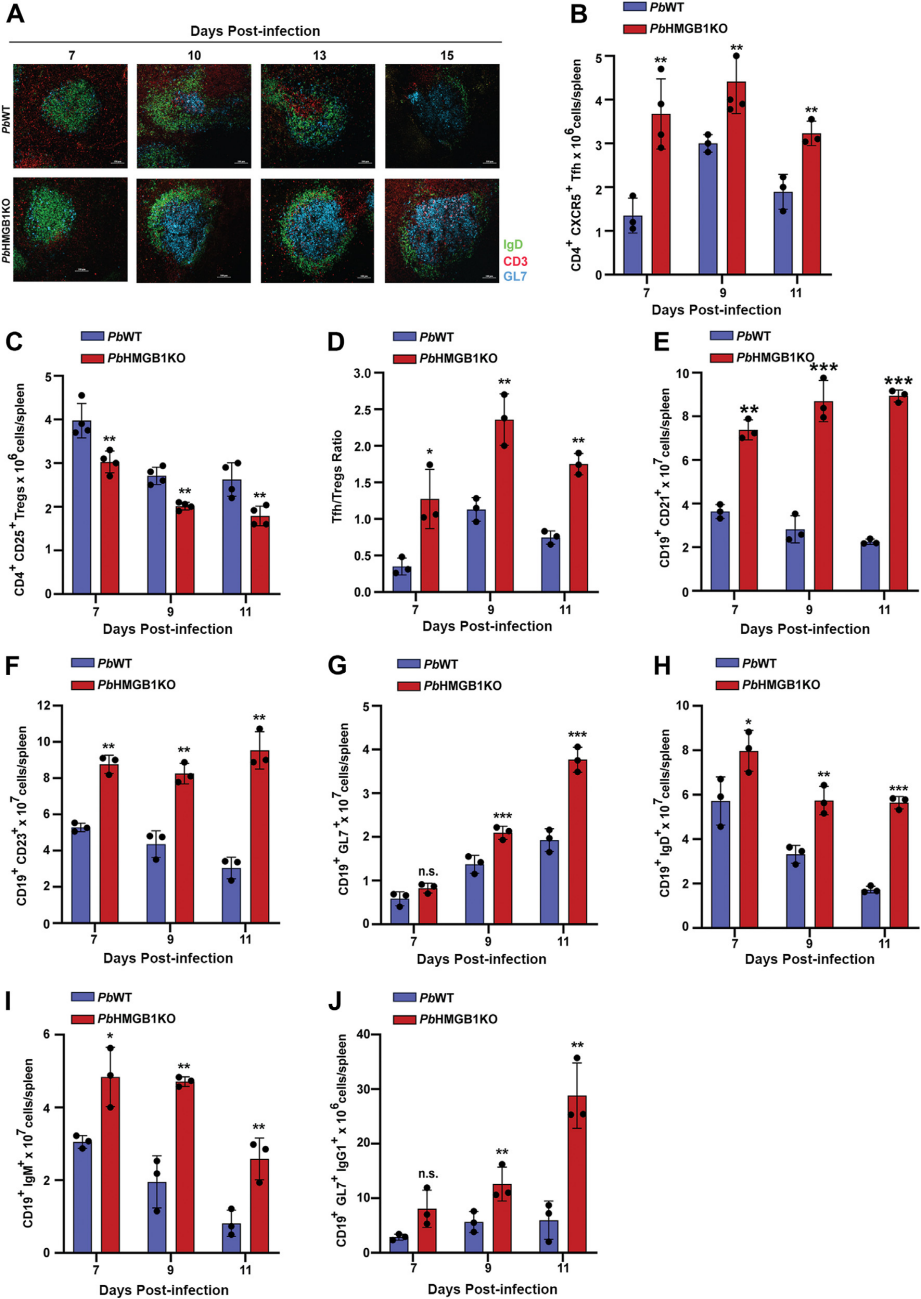
**Characterization of GC responses in *Pb*HMGB1KO-infected mice.***A*, immunofluorescence analyses of GCs in the spleen sections of *Pb*WT- and *Pb*HMGB1KO-infected mice. Images were captured using 20× objective. Scale bar = 50 μm. n = 3 independent experiments. *B*, Tfh in the spleen samples of *Pb*WT- and *Pb*-HMGB1KO-infected mice. *C*, tregs in the spleen samples. *D*, tregs/Tfh ratio in the spleen samples. The flow cytometry data (mean ± SD) represent at least three different mice for the respective days (∗*p* < 0.05, ∗∗*p* < 0.01; unpaired *t* test; two-tailed). *E–J*, B cell subtypes in the spleen samples of *Pb*WT- and *Pb*-HMGB1KO-infected mice. CD19^+^ CD21^+^ marginal zone B cells (*E*), CD19^+^ CD23^+^ follicular B cells (*F*), CD19^+^ GL^+^ GC B cells (*G*), IgD secreting CD19^+^ B cells (*H*), IgM secreting CD19^+^ B cells (*I*) and CD19^+^ GL^+^ GC B cells expressing IgG1 (*J*). The flow cytometry data (mean ± SD) represent three different mice (n.s.- not significant,∗*p* < 0.05, ∗∗*p* < 0.01, ∗∗∗*p* < 0.001; unpaired *t* test; two-tailed).

Germinal centers (GCs) offer the conducive niche for the interaction of T follicular helper cells with B cells leading to the proliferation and differentiation of B cells (34, 35). It has been shown that malaria infections can abrogate the germinal center responses leading to impaired protective immunity (36, 37, 38). Immunofluorescence analysis of the GCs in the spleen sections using GL7, IgD and CD3 antibodies that are specific for GCs, B cell follicles and T cell zones, respectively, suggested the restoration of GCs in the spleen of *Pb*HMGB1KO-infected mouse. While GCs were hardly detectable in the spleen of *Pb*WT-infected mice on day 7 to 15 post-infection, they were prominent in *Pb*HMGB1KO-infected mice with organized T cell zone and B cell follicles (Fig. 8*A*). Since T-follicular helper (Tfh) cells play an important role in determining GC responses and protective immunity, and regulatory T cells (Tregs) can impede the function of Tfh (37, 38), we examined the levels of CD4^+^ CXCR5^+^ Tfh and CD4^+^ CD25^+^ Tregs in *Pb*HMGB1KO-infected mice and determined the ratio of Tfh:Tregs. There was a significant increase in the number of Tfh (Figs. 8*B* and S6) with a concomitant decrease in Tregs (Figs. 8*C* and S6), and as a result, Tfh:Tregs ratio of *Pb*HMGB1KO-infected mouse spleen was ∼3 times higher when compared with *Pb*WT-infected mouse (Fig. 8*D*). There was also an increase in the marginal zone B cells (CD19^+^ CD21^+^), follicular B cells (CD19^+^ CD23^+^), IgM/IgD secreting B cells (CD19^+^ IgM^+^/IgD^+^), GC B cells (CD19^+^ GL^+^), and GC B cells expressing IgG1 (CD19^+^ GL^+^ IgG1) (Figs. 8, *E*–*J* and S7). These data suggested the robust GC responses of *Pb*HMGB1KO-infected mouse spleen.

### Long-term protection of *Pb*HMGB1KO-infected mice against *Pb*WT challenges

**Figure 9:**
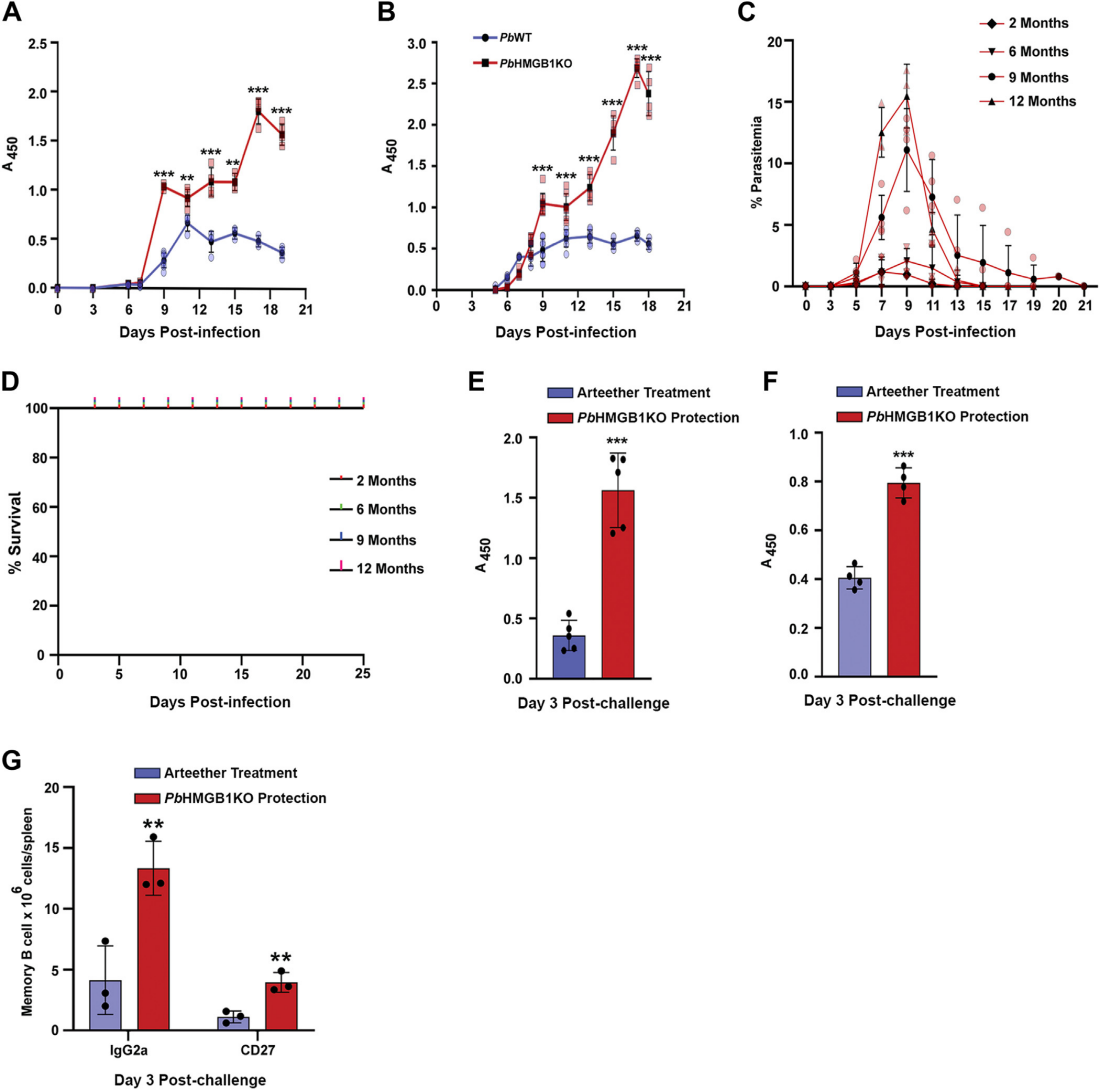
**Long-term protection of *Pb*HMGB1KO-infected mice.***A* and *B*, ELISA analyses of the sera collected from *Pb*HMGB1KO-infected mice against *Pb*WT parasite lysates (*A*) and iRBC lysates (*B*) to assess the parasite antigen-specific IgG. For control, sera collected from *Pb*WT-infected mice were used. The wells were coated with 100 μg of the total protein from parasite lysates or iRBC lysates. The data (mean ± SD) represent at least three different mice (∗∗*p* < 0.01, ∗∗∗*p* < 0.001; unpaired *t* test; two-tailed). *C*, blood parasitemia of *Pb*HMGB1KO-infected mice challenged with *Pb*WT parasites at 2, 6, 9 and 12 months post-protection. *D*, survival curves of the respective mice used for *Pb*WT challenge. The data (mean ± SD) represent at least three different mice for each time interval. *E* and *F*, assessment of parasite antigen-specific IgG in the sera of the protected mice infected earlier with *Pb*HMGB1KO parasites and challenged with *Pb*WT parasites. 100 μg of the total protein from *Pb*WT parasite lysates (*E*) and iRBC lysates (*F*) were used to coat the wells. Sera collected from day 3 post-challenge were used. For control, sera from *Pb*WT-infected mice cleared with a single dose of arteether (2 mg/mouse) and challenged with *Pb*WT parasites were used. The data (mean ± SD) represent at least three different mice (∗∗∗*p* < 0.001; unpaired *t* test; two-tailed). *G*, levels of CD27^+^ and IgG2a^+^ memory B cells isolated from the spleen of *Pb*HMGB1KO-infection protected mice on day 3 post-challenge. The flow cytometry data (mean ± SD) represent three different mice (∗∗*p* < 0.01; unpaired *t* test; two-tailed).

Since *Pb*HMGB1KO-infected mice could clear the blood parasitemia and exhibit better GC responses, we examined the levels of parasite antigen-specific IgG during the course of infection in *Pb*WT- and *Pb*HMGB1KO-infected mice. ELISA analyses were carried out by coating the wells individually with *Pb*WT parasite lysates (Fig. 9*A*) and infected RBC (iRBC) lysates (Fig. 9*B*). The iRBC lysates were included to represent the parasite antigens that are recruited on RBC membrane. In both the cases, parasite antigen-specific IgG levels were significantly higher in the sera collected from *Pb*HMGB1KO-infected mice in comparison with *Pb*WT-infected mice. In particular, the parasite antigen-specific IgG levels were at least 4 times higher in the sera collected during the clearance phase of *Pb*HMGB1KO-infected mice (Fig. 9, *A* and *B*). We then assessed the long-term protection of *Pb*HMGB1KO-infected mice against *Pb*WT parasite challenges at 2, 6, 9, and 12 months post-protection by challenging them intravenously with 10^5^ *Pb*WT parasites. Interestingly, all the mice were protected and the protection could last long even at 12 months (Fig. 9, *C* and *D*). Our next interest was to assess the memory response for which, we challenged *Pb*HMGB1KO-infected mice with intravenous administration of 10^6^ *Pb*WT parasites after 2 months of parasite clearance. For control, we used *Pb*WT-infected mice cleared with a single dose of arteether (2 mg/mouse) when the blood parasitemia was around ∼20%. It is known that the clearance of parasites in *Pb*WT-infected mice with antimalarials can lead to increased levels of parasite antigen-specific IgG and memory responses against the subsequent challenge with *Pb*WT parasites. On day 3 post-challenge, the sera were collected to assess the levels of the parasite antigen-specific IgG, and spleens were collected to examine the memory responses. Interestingly, the parasite antigen-specific IgG levels of *Pb*HMGB1KO-infected mice were at least three times higher in the case of wells coated with parasite lysates (Fig. 9*E*) and two times higher in the case of wells coated with iRBC lysates than the antimalarial-treated *Pb*WT-infected mice (Fig. 9*F*). This was also reflected in the increased levels of CD27^+^ and IgG2a^+^ memory B cells isolated from the spleen (Figs. 9*G* and S8). These results suggested that the clearance of blood parasitemia in *Pb*HMGB1KO-infected mice could lead to an efficient long-lasting memory response.

### *Pb*HMGB1 regulates *pir* expression

**Figure 10:**
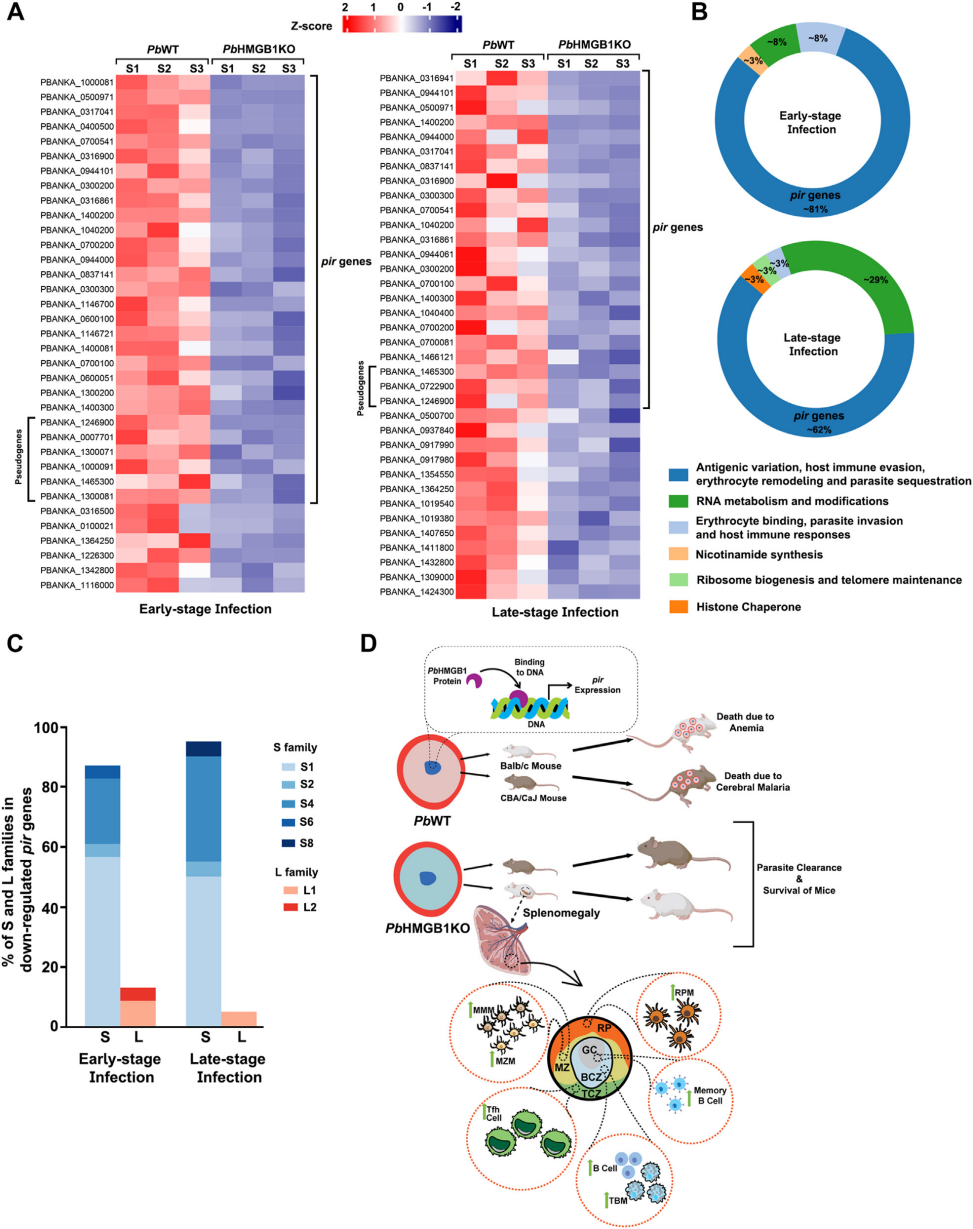
**Transcriptomics of *Pb*HMGB1KO parasites and schematic representation of the parasite clearance and long-lasting protective immunity in *Pb*HMGB1KO-infected mice.***A*, list of downregulated genes in *Pb*HMGB1KO parasites during early- and late-stage infections. The genes that showed significant down-regulation with greater than 1.5-fold change, FDR < 0.05 and adjusted *p*-value < 0.05 were considered (Benjamini-Hochberg procedure; multiple hypothesis testing). RNA-Seq analyses were carried out for three independent parasite pellets of *Pb*WT and *Pb*HMGB1KO parasites for early- and late-stage infections, respectively. *B*, donut chart representing the gene ontologies of significantly down-regulated genes based on the functional annotations available in PlasmoDB and published literature. *C*, percentage of S and L families in the down-regulated *pir* genes and the proportion of various clades. The entire details of RNA-Seq analyses are provided in Dataset S1. *D*, model depicting the role of *Pb*HMGB1 in regulating the expression of *pir* genes and the splenic events leading to parasite clearance and long-lasting protective immunity in *Pb*HMGB1KO-infected mice. The protection from anemia and cerebral malaria mortality due to the clearance of *Pb*HMGB1KO parasites in Balb/c and CBA/CaJ mice is shown. The preservation of splenic architecture with effective germinal center responses in *Pb*HMGB1KO-infected mice is represented. BCZ, B cell zone; GC, germinal center; MZ, marginal zone; MMM, marginal metallophilic macrophages; MZM, marginal zone macrophages; RP, red pulp; RPM, red pup macrophages; TBM, tingible body macrophages; TCZ, T cell zone.

Transcriptomic studies were carried out to examine the reasons behind the self-clearance phenotype of *Pb*HMGB1KO parasites, and the lack of cerebral pathogenesis and long-term protection observed for *Pb*HMGB1KO-infected mice. For this, RNA-Seq analyses were performed for *Pb*WT and *Pb*HMGB1KO parasites isolated from early-stage (day seven-eighths post-infection) and late-stage (day 12–15 post-infection) infections. Despite having severe growth limitations and self-clearance phenotype, *Pb*HMGB1KO parasites showed a significant down-regulation for only a small repertoire of genes. Interestingly, of the total number of 36 genes that were down-regulated in the early-stage infections, ∼81% of them represented *pir* gene family that is known to be associated with the antigenic variation, host immune evasion, erythrocyte remodeling, and parasite sequestration (Fig. 10, *A* and *B* and Dataset S1). A similar pattern was also observed for the late-stage infections wherein, ∼62% of the total 37 genes down-regulated belonged to *pir* gene family (Fig. 10, *A* and *B* and Dataset S1). Within the down-regulated *pir* genes, ∼41% and ∼26% of them were unique for early- and late-stage infections, respectively, suggesting the dynamic changes in *pir* gene expression during the course of blood-stage infections. In particular, ∼87% of the early-stage and ∼95% of the late-stage down-regulated *pir* genes represented the short (S) family of S1, S2, S4 S6, and S8 clades, and almost all of them are expressed in the blood stages (39). The contribution of the long (L) family to the downregulated *pir* genes was strikingly low (Fig. 10*C* and Dataset S1). The rest of the downregulated genes included fam proteins, small nucleolar RNAs, and other proteins that are associated with RNA modifications and metabolism, and ribosomal functions. The down-regulation of small nucleolar RNA genes appeared to be more pronounced in late-stage infections (Fig. 10*B* and Dataset S1).

The analysis of up-regulated genes in *Pb*HMGB1KO parasites suggested significant changes in the transcript levels of 65 genes for early-stage and 54 genes for late-stage infections. There was also an upregulation of a few *pir* genes that represented ∼30% of the total genes. Further, ∼50% of the early- and late-stage up-regulated *pir* genes were pseudogenes in contrast to ∼20% that was observed for the down-regulated *pir* genes (Fig. S9, *A* and *B* and Dataset S1). Of the 9 *pir* genes that were up-regulated in early- and late-stage infections, two-third belonged to the S family (Fig. S9*C* and Dataset S1). Interestingly, there was a prominent increase in the percentage of fam family genes and other genes that encode reticulocyte binding proteins, erythrocyte membrane antigen 1, and exported proteins. These proteins play important role in erythrocyte binding, parasite invasion, and host immune responses, and they represented ∼50% of the up-regulated genes in the early- and late-stage infections of *Pb*HMGB1KO parasites (Fig. S9*B* and Dataset S1). It is also worthwhile to mention that there were no significant changes in the transcript levels of the other three HMGBs - HMGB2, HMGB3, and HMGB4 in *Pb*HMGB1KO parasites (Dataset S1). All these data suggested that *pir* gene expression is altered in *Pb*HMGB1KO parasites displaying a prominent down-regulation with a concomitant up-regulation of genes that are involved in erythrocyte binding and parasite invasion.

## Discussion

HMGB proteins are ubiquitous non-histone proteins playing important nuclear functions such as regulation of gene expression, DNA replication and repair, telomere homeostasis, and chromosome stability (14, 15, 16, 17, 40, 41). While human and mouse HMGB1 have been extensively studied (22, 29), there are also quite a few studies on HMGB2 and other HMGB proteins (42, 43, 44, 45). In addition to the nuclear functions, HMGB1 regulates autophagy and apoptosis by shuttling between the nucleus and cytosol (18, 19, 46). It is also actively secreted into the extracellular space by the activated immune cells or passively released during necrosis to function as a DAMP/cytokine. The interaction of extracellular HMGB1 with receptors such as RAGE, TLR4, and other surface molecules can trigger various downstream signaling events that activate MyD88, nuclear factor κB, MAPK, phosphatidylinositol3-kinase (PI3K), and IFN regulatory factor pathways (20, 22, 47). All these eventually render HMGB1 performing a plethora of functions that include cell differentiation, proliferation, migration and senescence, metastasis, angiogenesis, inflammation, and immune responses (22, 48). The present study highlights that *Plasmodium* HMGB1 lacks the characteristic features of mammalian HMGB1. It has been shown that the disulfide bond formation between Cys23 and Cys45 present in box A is required for the translocation of mouse and human HMGB1 from the nucleus to the cytosol regulating autophagy, and for the extracellular release. Interestingly, the entire box A is absent in the parasite HMGB1. Similarly, Cys106 in box B is essential for the nucleocytoplasmic translocation, and proinflammatory and immunomodulatory functions of mouse and human HMGB1 (20, 22, 29, 30, 49). Although the parasite HMGB1 has retained box B, it lacks the corresponding Cys residue and contains Ala instead of Cys.

We show that the parasite HMGB1 does not exhibit TNF-α stimulatory activity and the TNF-α stimulatory domain of box B present in the parasite HMGB1 has undergone extensive modifications. The synthetic peptide of *Pb*HMGB1 corresponding to the TNF-α stimulatory domain of mammalian HMGB1 and the r*Pb*HMGB1 do not induce TNF-α release in the murine macrophage cell line. Further, the mutation of Ala to Cys in box B is inadequate to restore the TNF-α stimulatory activity of r*Pb*HMGB1. Our results from site-directed mutagenesis studies suggest that eight independent mutations are additionally required in box-B of the parasite HMGB1 to show similar levels of TNF-α stimulatory activity as mouse HMGB1. An earlier study alluded to the proinflammatory activity of parasite HMGB1 by performing *in vitro* TNF-α release assays with r*Pf*HMGB1 and showing the substantial release of TNF-α at higher concentrations (100–300 μg/ml) of r*Pf*HMGB1 in contrast to mouse HMGB1 that could exhibit TNF-α release at a concentration of 0.4 to 2.5 μg/ml. Further, r*Pf*HMGB1 was not subjected to DNase treatment, and polymyxin B was added along with r*Pf*HMGB1 while performing the assays (50). In the present study, r*Pb*HMGB1 is subjected to DNase treatment and high-capacity endotoxin removal before performing the *in vitro* assays. The results are also verified with the synthetic peptides corresponding to TNF-α stimulatory domain and mutagenesis studies. Our findings lay the platform to examine the TNF-α stimulatory activity of HMGBs from other apicomplexan parasites. A comparison of HMGBs from the closely related non-parasitic (*Chromera* and *Vitrella*) and parasitic alveolates suggests sequence homology with the TNF-α stimulatory domain of mammalian HMGBs, although there are considerable changes as observed for *Plasmodium* HMGB1 (Fig. S10). Interestingly, Cys106 of mammalian HMGB1 is conserved in HMGBs of *Vitrella*, *Haemoproteus*, and *Cryptosporidium*, but not in *Chromera* and other apicomplexans. There are also additional HMG box-containing proteins present and further studies are required to address whether the loss of TNF-α stimulatory activity is a common feature of apicomplexan HMGBs.

In agreement with the lack of TNF-α stimulatory activity and the three Cys residues that are essential for extracellular functions, *Pb*HMGB1 is undetectable in the plasma of *Pb*-infected mice. Extracellular mouse/human HMGB1 serves as a potent proinflammatory cytokine and it is associated with systemic inflammation like sepsis and various other inflammatory diseases (21, 51, 52). The parasitized-RBCs encounter numerous host immune cells and are phagocytosed extensively by monocytes, macrophages, and dendritic cells present in the circulation and organs like spleen, liver, and lungs (2, 53, 54). This, in turn, may lead to the exposure of immune cells to the parasite HMGB1 and if the parasite HMGB1 is proinflammatory, such exposure may trigger abrupt immune signaling events resulting in an uncontrolled inflammation that will be deleterious to the host. This is also true for the release of parasite HMGB1 which may happen during the clearance of parasitized-RBCs in the spleen. Hence, the loss of extracellular and proinflammatory functions of parasite HMGB1 might be advantageous in the context of prolonged host survival that is required for the successful transmission to the definitive hosts - *Anopheline* mosquitoes.

Another interesting finding is the deletion of HMGB1 in a lethal rodent parasite strain, *Pb* ANKA, leading to complete protection from mortality due to anemia and cerebral malaria (Fig. 10*D*). *Pb*HMGB1KO parasites are completely cleared when the blood parasitemia reaches around 20%. The ability of *Pb*HMB1KO parasites collected from the clearance phase to induce fresh infections in naïve mice suggests the absence of growth defects in the parasite, and the protection is mediated by host immune responses. Splenic macrophages play an important role in eliminating the parasitized RBCs and the control of blood parasitemia (55, 56, 57). *Pb*HMGB1KO-infected mice display a significant increase in the red pulp, marginal zone, and white pulp macrophages. This is also true for the cDCs that are involved in T cell priming. However, the levels of pDCs do not seem to differ between the spleen samples of *Pb*WT- and *Pb*HMGB1KO-infected mice. More importantly, the splenic architecture and GCs are well-preserved in *Pb*HMB1KO-infected mice. This is in stark contrast to the *Pb*WT-infected mice with disease severity wherein, a profound disorganization of splenic architecture and germinal centers is observed. In the context of GC reactions being crucial for adaptive immune responses and generation of parasite-specific antibodies (36, 37, 58), *Pb*HMGB1KO-infected mice show a robust parasite-specific antibody response. There is also an increase in the marginal zone, follicular, and GC B cells (Fig. 10). In particular, Tfh cells essential for GC formation, affinity maturation, and generation of high-affinity antibodies and memory B cells are significantly higher in the *Pb*HMB1KO-infected mouse spleen. A number of studies with rodent and human infections have highlighted the importance of Tfh cells in the development of functional antibodies and long-lasting immunity against malaria (37, 38, 59, 60, 61). In agreement with these results, mice recovered from *Pb*HMGB1KO infections display long-lasting immunity and protection when re-challenged with *Pb*WT parasites. Altogether, our *in vivo* findings emphasize the important role of *Pb*HMGB1 in modulating the host splenic responses. It is worthwhile to mention that the phenotype of *Pb*HMGB1KO differs significantly from HMGB2 deletion. While HMGB2 deletion in *Pb*NK65 strain could lead to self-clearance and protection in infected mice, its deletion in highly lethal *Pb*ANKA or *Py*YM strains leads to 100% mortality in infected mice with a slight attenuation of asexual stage growth (24, 25, 26). Further, microarray studies carried out for blood-stage parasites have suggested the prominent role of *Py*HMGB2 in regulating the genes expressed in gametocytes that remain translationally repressed until the onset of mosquito stages (26). *Py*HMGB2KO parasites display a significant reduction in oocyst and sporozoite formation, and this has also been shown for HMGB2 deletion in *Pf* parasites (27). It is possible that the function of HMGB2 is more confined to the parasite development in the mosquitoes.

Intrigued by the absence of TNF-α stimulatory activity and extracellular release, we have examined the gene regulatory function of *Pb*HMGB1 for the reasons behind robust splenic responses, self-clearance, and protection phenotype in *Pb*HMGB1KO-infected mice. Chromosome conformation capture and transcriptome analyses of *in vitro* cultured *Pf* parasites lacking HMGB1 have suggested the loss of centromere/telomere organization resulting in the silencing of *var* genes that are associated with parasite sequestration, disease pathogenesis, and host immune evasion (28). However, *var* genes are present only in the *Plasmodia* species of *Laverania* subgenus such as *Pf* and *Plasmodium reichenowi* (62). Other non-*Laverania* species infecting humans (*vivax*, *malariae*, *ovale* and *knowlesi*), non-human primates (*cynomolgi*, *coatneyi etc.*) and rodents (*berghei*, *yoelii etc.*) have *Plasmodium* interspersed repeat (*pir*) multigene families with suggested similar roles as *var*. It is also known that unlike the mutually exclusive expression of one *var* gene at a time, multiple *pir* genes are expressed together in various stages of the parasite life cycle (63, 64, 65, 66). Our findings suggest that the deletion of *Pb*HMGB1 leads to a prominent down-regulation of *pir* genes that are expressed in the blood stages (Fig. 10*D*). Interestingly, a larger proportion of these down-regulated *pir* genes belong to S family and further ChIP-Seq studies are required to gain insights on the promoter occupancy of *Pb*HMGB1 and other co-regulators. The down-regulation of small nucleolar RNAs in the late-stage infections of *Pb*HMGB1KO parasites is also noteworthy and it could be a reflection of the decreased *pir* expression. With increasing evidence on the functions of small nucleolar RNAs in RNA modifications, mRNA abundance, and translational regulation, it would be of interest to examine their role in regulating the *pir* expression. There is also a small subset of *pir* genes that are upregulated in *Pb*HMGB1KO parasites and it is not clear at this stage whether their expressions are controlled by other HMGBs or transcription factors. Another prominent aspect is the upregulation of fam, erythrocyte binding, and exported proteins involved in host receptor interactions, parasite invasion, and immune responses. Such processes might help *Pb*HMGB1KO parasites to deploy compensatory mechanisms for sustaining the blood stage growth and overcoming splenic clearance. There is also a scope for understanding the significance of HMGB1 in the other stages of the *Plasmodium* life cycle. In summary, the present study highlights the gene regulatory function of parasite HMGB1 and its significance in modulating disease pathogenesis, splenic responses, and host immune evasion in blood-stage infections. Such insights would help us to understand the virulence and immune evasion mechanisms of malaria parasites, and explore the possibility of developing a genetically attenuated blood-stage vaccine for malaria if HMGB1 deletion can lead to self-clearance and protection for human parasites.

## Experimental Procedures

### Multiple sequence alignment of HMGB1

The HMGB1 sequences of mouse (NP_034569.1) and human (CAG33144.1) were retrieved from NCBI (https://www.ncbi.nlm.nih.gov/), and the sequences of *Pb* (PBANKA_0601900), *Pf* (PF3D7_1202900), *Plasmodium vivax* (PVP01_1302200), *Plasmodium ovale* (PocGH01_13013100), *P malariae* (PmUG01_13013200), *Plasmodium knowlesi* (PKA1H_130007700), *P. reichenowi* (PRCDC_1202300), *Plasmodium cynomolgi* (PcyM_1302700), *P. yoelii* (PY17X_0604400) and *Plasmodium chabaudi* (PCHAS_0603700) were retrieved from PlasmoDB (https://plasmodb.org/plasmo/app). Multiple sequence alignment was carried out with SeaView Version 5.0.5 (https://doua.prabi.fr/software/seaview) using MUSCLE (Multiple Sequence Comparison by Log-Expectation) algorithm.

### Expression and purification of r*Pb*HMGB1, r*Pb*HMGB1^C41^, r*Pb*HMGB1^5mut^, r*Pb*HMGB1^9mut^ and rmHMGB1

The cDNA sequences of *Pb*HMGB1 and mouse HMGB1 (BC110667.1) were retrieved from PlasmoDB (https://plasmodb.org/plasmo/app) and NCBI GenBank (https://www.ncbi.nlm.nih.gov/), respectively. RNeasy Mini Kit (Qiagen, 74104) was utilized for the isolation of total RNA from *Pb* parasites and mouse liver according to the manufacturer’s protocol. RevertAid Reverse Transcriptase (Thermo Fisher Scientific, EP0442) was used for the synthesis of cDNA from 1 μg of total RNA followed by PCR amplification with Phusion High-Fidelity DNA Polymerase (New England Biolabs, M0530). The following are the forward and reverse primers used for *Pb*HMGB1: 5′-GCCAGGAGCTCATGGATGGCATGAAAAAATTTAAAGATATGAAAATGGGTG-3′ and 5′-GCCAGGAATTCTTATTTCATTTTACTTTTGGCATATTCCATTTTTTCTTTT-3′. *SacI* and *EcoRI* restriction sites used for cloning are underlined. The following are the forward and reverse primers used for mouse HMGB1: 5′-GCCAGGATCCATGGGCAAAGGAGATCCTAAGAAGCCGAG-3′ and 5′-GCCCGGTACCTTATTCATCATCATCATCTTCTTCTTCATC-3’. *BamHI* and *KpnI* restriction sites are underlined. The PCR products were subjected to digestion with the respective restriction enzymes and the digested products were cloned in-frame into pRSETA plasmid (Thermo Fisher Scientific) having 6xHis tag at the N-terminus region. Protein expression was carried out in *E. coli* Rosetta2DE3pLysS strain (Novagen). In brief, *E. coli* Rosetta2DE3PLysS strain transformed with recombinant plasmid was grown to an A_600_ of 0.8 at 30 °C. The protein induction was carried out using 1 mM isopropyl-β-D-thiogalactoside (IPTG) (MP Biomedicals, 11IPTG0001) at 18 °C for 12 h. The overexpressed recombinant (r) *Pb*HMGB1 or mouse HMGB1 (mHMGB1) with N-terminal histidine tag was purified under identical conditions using Ni^2+^-NTA agarose resin (Qiagen, 30210). For this, the respective bacterial cell pellet expressing the recombinant protein was resuspended in lysis buffer containing 50 mM Tris pH 8.0, 500 mM NaCl, 2% glycerol, 0.5% Triton X-100 and protease inhibitors, sonicated and centrifuged at 43,000*g* for 60 min. The supernatant was collected and loaded onto a column packed with Ni^2+^-NTA resin, and the column was washed consecutively with lysis buffer containing 5 and 50 mM imidazole. The recombinant protein was eluted with lysis buffer containing 100 mM imidazole. The purified protein was then dialyzed against 25 mM Tris pH 8.0 containing 100 mM NaCl and 2% glycerol, followed by further purification by S-Sepharose cation exchange chromatography (Macro-Prep High S Media, Bio-Rad, 1560030). The dialyzed recombinant protein was loaded onto a column packed with S-Sepharose resin and the column was washed consecutively with 25 mM Tris buffer pH 8.0 containing 125 mM and 250 mM, respectively, followed by elution in the same buffer containing 500 mM NaCl. The purified recombinant protein was subjected to enterokinase (New England Biolabs, P8070S) cleavage to remove the 6xHis tag and Xpress epitope, followed by DNase I (New England Biolabs, M0303S) treatment (20 Units for 1 mg of recombinant protein) to remove any bacterial genomic DNA contaminants. The resultant protein was then loaded on Pierce High Capacity Endotoxin Removal Resin (88,270) (1 ml of resin for 1 mg of recombinant protein) to remove LPS contamination as per the manufacturer’s protocol. The primers used to perform the site-directed mutagenesis for generating - r*Pb*HMGB1^C41^, r*Pb*HMGB1^5mut,^ and r*Pb*HMGB1^9mut^ plasmids are provided in Table S1. PCR amplification was carried out with the respective primers and the amplified products were subjected to *DpnI* (New England Biolabs, R0176S) digestion to remove the recombinant plasmid used as a template, followed by transformation into *E. coli* NovaBlue (Novagen) bacterial strain. The plasmid isolation was carried out for the individual recombinant colonies and the presence of respective mutation was confirmed by DNA sequencing using Sanger’s method. For the generation of r*Pb*HMGB1^C41^, r*Pb*HMGB1 plasmid was used as a template. For the subsequent mutations, the mutant plasmid generated for the previous mutation was used as a template for the next mutation. The mutant proteins - r*Pb*HMGB1^C41^, r*Pb*HMGB1^5mut,^ and r*Pb*HMGB1^9mut^ were also purified and processed following identical conditions.

### TNF-α stimulatory activity of synthetic peptides and recombinant proteins

Murine macrophage-like Raw 264.7 cell line was cultured in DMEM medium (Gibco 12,100,038, Thermo fisher) containing 10% FBS and 1% penicillin-streptomycin (v/v) (15,140–122 Gibco Thermo fisher) under 5% CO_2_ at 37 °C and 95% humidity (30). 2 x 10^4^ cells were seeded and treated for 12 h with different concentrations of synthetic peptides and recombinant proteins. The 20-mer peptide of mouse HMGB1 (FKDPNAPKRPPSAFFLFCSE) and *Pb*HMGB1 (KKDPHAPKRSLSAYMFFAKE) was purchased from GL Biochem (Shangai) Ltd, with greater than 90% purity as confirmed by HPLC. The respective peptides were dissolved in DMSO and diluted further with sterile PBS to achieve the working stocks as per the manufacturer’s instruction. The recombinant protein concentrations were estimated using the Pierce BCA Protein Assay Kit (23,225). The culture supernatants collected after 12 h were centrifuged at 5000*g* for 5 min at 4 °C to remove the cell debris, if any. The resultant supernatants were used to perform ELISA for estimating the levels of TNF-α released using TNF alpha Mouse ELISA Kit (ab46105) as per the manufacturer’s protocol. The culture supernatants of the cells treated with PBS containing DMSO or buffer containing 25 mM Tris pH 8.0 and 500 mM NaCl were used as negative controls for the background subtraction. TNF-α release induced by LPS (10 μg/ml) treatment was used as a positive control in all the experiments.

### Generation of *Pb*WT^*HMGB1-GFP*^ transgenic parasites

To generate *Pb*WT^*HMGB1-GFP*^ transgenic parasites, a 736 bp upstream promoter sequence along with the entire coding sequence of *Pb*HMGB1 was amplified using Phusion DNA polymerase with the following forward and reverse primers: 5′- GCCACCGCGGCGGTTTATTTTGGCAAAATTAAAAGGG-3′ and 5′-GCAAGGATCC**TCCAGCACCAGCAGCAGCACC**TTTCATTTTACTTTTGGCATATTCCAT-3′. *SacII* and *BamHI* restriction sites are underlined and the 21 bp linker sequence included in the reverse primer is highlighted in bold. The amplified fragment of 1076 bp was cloned in-frame upstream to the GFP sequence of *pL0031* plasmid (67). The cloned fragment with in-frame GFP was then amplified with the forward primer and a GFP-specific reverse primer: 5′-GCAAGAATTCTTATTTGTATAGTTCATCCATGCCATG-3′. *EcoRI* restriction site in the GFP-specific reverse primer is underlined. The resultant fragment of 1795 bp was cloned into the GOMO-GFP-luciferase plasmid (68, 69) by replacing the GFP-luciferase sequence. To perform double-crossover recombination, a 3′UTR sequence of *Pb*HMGB1 (656 bp) was amplified using the forward and reverse primers: 5′-GCCACTCGAGGGAAAGTATATATAATAAAATATTATATGAATGTG-3′ and 5′-GCCCGGTACCGAACGTGCTAAAATAACACCA-3′, and cloned into the afore-mentioned plasmid. *XhoI* and *KpnI* restriction sites are underlined. The recombinant plasmid was then digested with *SacII* and *KpnI* restriction sites and the released fragment was transfected into *Pb*WT schizonts using 4D-Nucleofector (Lonza). The transfected parasites were then injected intravenously into naïve Balb/c mice and selected using pyrimethamine (70 μg/ml) in drinking water. The clonal selection was performed by limiting dilution and the transgenic *Pb*WT^*HMGB1-GFP*^ parasites obtained were then subjected to 5-fluorocytosine treatment in drinking water (1 mg/ml) to remove the mCherry reporter cassette (70) The site-specific integration in *Pb*WT^*HMGB1-GFP*^ transgenic parasites was confirmed using a forward primer designed upstream to the promoter sequence of *Pb*HMGB1 used for transfection (5′-ACGGATATATATATAAGAAAATCGCATTTAAATATG-3′) and GFP-specific reverse primer.

### Localization of *Pb*HMGB1

Live imaging of GFP fluorescence in *Pb*WT^*HMGB1-GFP*^ parasites expressing *Pb*HMGB1-GFP fusion protein was carried out as described (71, 72). Balb/c mice were infected intraperitoneally with 10^5^ *Pb*WT^*HMGB1-GFP*^-parasitized RBCs and when the blood parasitemia reached around 5% on day 6, 10 μl of tail vein blood was collected in heparinized PBS. The blood was then centrifuged to remove the plasma and the cells were washed twice with PBS. The cells were then resuspended in PBS containing DAPI (1 μg/ml) and incubated at room temperature for 20 min, followed by three washes with PBS. The cells containing iRBCs were then resuspended in PBS and transferred to glass-bottom Petri dishes for live imaging. Indirect immunofluorescence analysis of *Pb*HMGB1-GFP localization in *Pb*WT^*HMGB1-GFP*^ parasites was carried out as described earlier (71). In brief, the tail vein blood collected from the infected mice was washed thrice with PBS and overlaid on poly-L-lysine coated coverslip for 1 h, followed by fixing with 4% paraformaldehyde in PBS (w/v) containing 0.0075% glutaraldehyde (v/v) for 30 min at room temperature. The cells were permeabilized with 0.1% Triton X-100 (v/v) in PBS for 10 min, treated with 0.1 M glycine for 10 min and subsequently blocked with 2% BSA (w/v) for 3 h at room temperature. The cells were then incubated with rabbit polyclonal anti-GFP antibody (Abcam; ab290; 1:500 dilution) for 4 h at room temperature, washed with PBS, and then treated with FITC-conjugated donkey anti-rabbit IgG (SantaCruz, sc-2090; 1:200 dilution) for 2 h at room temperature. After staining with DAPI (1 μg/ml in PBS) for 20 min and subsequent washing with PBS, the coverslip was dried and mounted on a glass slide with ProLong TM Gold antifade (Invitrogen). The localization of *Pb*HMGB1 in *Pb*WT parasites using polyclonal anti-r*Pb*HMGB1 antibodies (1:1000 dilution) was also carried out in a similar fashion. All the images were captured with 100X objective using an Olympus IX83 inverted microscope having a high-performance camera (DP73) and processed using Olympus CellSens Dimension software.

### ELISA for the extracellular presence of HMGB1

To examine the extracellular presence of *Pb*HMGB1-GFP, whole blood samples were collected form the mice infected with *Pb*WT^*HMGB1-GFP*^ parasites through cardiac puncture using heparin as an anticoagulant. The blood samples were then centrifuged at 2000*g* at 4 °C and the separated plasma samples were used to coat the wells of ELISA plate in 50 mM carbonate buffer pH 9.6 for overnight at 4 °C. After blocking with 3% BSA (w/v) in PBS containing 0.05% (v/v) Tween 20 (PBST) at room temperature for 2 h, the wells were treated for 3 h with rabbit polyclonal anti-GFP antibody (Abcam; ab290; 1:3000 dilution) and washed thrice with PBST, followed by 2 h treatment with peroxidase-conjugated goat anti-rabbit IgG antibody (ab97051; 1:25,000 dilution). After washing thrice with PBST, 3,3′,5,5′-tetramethylbenzidine (TMB) liquid substrate was added, followed by stopping with 1 N H_2_SO_4_ and measuring the absorbance at 450 nm. For control and spiking the plasma samples, *Pb*WT and *Pb*WT^*HMGB1-GFP*^ parasite lysates were prepared by resuspending the respective parasite pellets in 50 mM Tris pH 7.5 containing 100 mM NaCl, 0.05% Tween 20 (Sigma-Aldrich) and 1× Halt Protease Inhibitor Cocktail (Thermo Fisher Scientific), followed by sonication in ice. The lysates were then centrifuged at 13,000*g* for 15 min at 4 °C and the supernatants collected were used to coat the wells of ELISA plate or for spiking the plasma samples after estimating the total protein content. For examining the presence of *Pb*HMGB1 in the plasma samples of *Pb*WT-infected mice and *Pb*WT parasite lysates using polyclonal anti-r*Pb*HMGB1 antibodies, a high-sensitivity method was followed (73) wherein, perchloric acid addition to the samples was carried out to the final concentration of 3% (v/v), followed by centrifugation at 15,000*g* for 5 min at 4 °C. The supernatants collected were then neutralized with 1.5 N NaOH and used to coat the wells of ELISA plate. Polyclonal anti-r*Pb*HMGB1 antibodies were used at 1:3000 dilution.

### Generation of *Pb*HMGB1KO and *Pb*HMGB1KO^*Luc*^ parasites

To generate *Pb*HMGB1KO parasites, 5′UTR region of *Pb*HMGB1 was amplified from *Pb*WT genomic DNA using the following forward and reverse primers: 5′-GCCAGGGCCCCGGTTTATTTTGGCAAAATAAAAGGG-3′ and 5′-GCCAGATCTAGAAAACCTTTTTTTTTAAAATATAAAGGACAGG-3’. *ApaI* and *XbaI* restriction sites are underlined. Similarly, 3′UTR region of *Pb*HMGB1 was amplified using the following forward and reverse primers: 5′-GCCAGGTACCGGAAAGTATATATAATAAAATATTATATGAATGTG-3′ and 5′-GCCCGCGGCCGCGAACGTGCTAAAATAACACCA-3′. *KpnI* and *NotI* restriction sites are underlined. The resultant 5′UTR and 3′UTR fragments were digested with respective restriction enzymes and cloned into *pL0006* plasmid on either side flanking the human DHFR selection cassette (68). The recombinant plasmid was then digested with *ApaI* and *NotI*, and the released fragment was transfected into *Pb*WT schizonts using 4D-Nucleofector (Lonza). The transfected parasites were then injected intravenously into naïve mice and selected using pyrimethamine. The pyrimethamine-resistant parasites were subjected to clonal selection by limiting dilution. The protective phenotype of *Pb*HMGB1KO parasites was confirmed with two independent clones. To generate *Pb*HMGB1KO^*Luc*^ parasites, a similar set of primers were used except for the changes in the restriction sites. For 5′UTR forward and reverse primers, *SacII* and *NotI* restriction sites were used. For 3′UTR forward and reverse primers, *XhoI* and *KpnI* restriction sites were used. The digested 5′UTR and 3′UTR fragments were cloned into GOMO-GFP-Luc plasmid on either side flanking GFP-Luc expressing cassette and a drug selection cassette expressing hDHFR fused with yFCU (yeast cytosine deaminase-uridyl phosphoribosyl transferase) and m-Cherry (69). After digesting the recombinant plasmid with *SacII* and *KpnI*, transfection was carried out as described for *Pb*HMGB1KO parasites. For control in bioluminescence studies, *Pb*Control^*Luc*^ parasites reported in our earlier studies were used (70).

### Genetic complementation of *Pb*HMGB1 in *Pb*HMGB1KO^*Luc*^ parasites

For genetic complementation, *Pb*HMGB1KO^*Luc*^-infected mice were treated with 5-fluorocytosine (1 mg/ml) to remove h*DHFR*-y*FCU* selection gene cassette (68, 69) and the resultant marker-free *Pb*HMGB1KO^*Luc*^ parasites were confirmed by the loss of m-cherry fluorescence. The parasites were then transfected with GOMO-GFP-Luc plasmid, wherein the *Pb*eIF1α promoter and the downstream luciferase were replaced with the *Pb*HMGB1 gene under its endogenous promoter. The corresponding sequence of −736 to +318 bp was PCR amplified with the following forward and reverse primers: 5′-GCCACCGCGGCGGTTTATTTTGGCAAAATTAAAAGGG-3′ and 5′-GCAAGAATTCTTATTTCATTTTACTTTTGGCATATTCCATTTTTTCTTTT-3′. *SacII* and *EcoRI* restriction sites used for cloning are underlined. The 3′UTR fragment of *Pb*HMGB1 was cloned using *XhoI* and *KpnI* restriction sites. The recombinant plasmid was then digested with *SacII* and *KpnI* and transfection was carried out as described earlier. The site-specific integration in the *Pb*HMGB1KO^*+HMGB1*^ parasites was confirmed using a forward primer 5′-ACGGATATATATATAAGAAAATCGCATTTAAATATG-3′ designed upstream to the promoter sequence of *Pb*HMGB1 used for transfection and a *Pb*DHFR specific reverse primer 5′- TAGCTAAAATTATGAACATTTTATTTTTTGTTCAGAAAAAA-3′.

### *P. berghei* infection studies

All the mice studies were carried out with the approval (ILS/IAEC-114-AH/APR-18) of the Institutional Animal Ethics Committee (IAEC) of the Institute of Life Sciences, Bhubaneswar, as per the national guidelines of “The Committee for the Purpose of Control and Supervision of Experiments on Animals (CPCSEA)”. The mice were bred at the animal house facility of the Institute of Life Sciences and maintained under the standard conditions of 25 °C and 40 to 50% relative humidity with a periodic 12 h light/dark cycle and *ad libitum* feed and water. *P. berghei* (*Pb*) ANKA strain was propagated in 7 to 8 weeks old male/female Balb/c mice and the asexual stage infections were initiated by injecting parasitized RBCs into the naïve mice through the intraperitoneal route. The deletion of *Pb*HMGB1, and the generation of *Pb*HMGB1KO, *Pb*HMGB1KO^*Luc*^, and *Pb*WT^*HMGB1-GFP*^ parasites were performed in the *Pb*ANKA strain. For growth analyses of *Pb*WT and transgenic parasites, assessment of protective phenotype in *Pb*HMGB1KO-infected mice, and challenging experiments, the required number of parasitized RBCs for the respective parasites were injected through intraperitoneal/intravenous route in Balb/c mice. Giemsa-stained smears were routinely prepared from the tail vein blood of the infected mice to assess the blood parasitemia (70). For the collection of spleen samples to perform immunohistochemical analyses and splenocyte isolations, infected mice were subjected to transcardial perfusion with PBS on the respective days. For ECM experiments, 7 to 8 weeks old male/female CBA/CaJ mice were used for the propagation of the respective parasites and for the subsequent ECM analyses.

### Histological analyses

To perform H&E staining of the spleen sections, *Pb*WT- and *Pb*HMGB1KO-infected mice were subjected to transcardial perfusion with PBS. The spleens were then dissected out and formalin-fixed for 72 h, followed by serial dehydrations with ethanol and clearing with xylene for 1 h. The paraffin blocks were then prepared and tissue sections of 5 μm thickness were generated using Leica RM2125RT rotary microtome. The sections were deparaffinized and serially hydrated before staining with hematoxylin and eosin (70). For immunohistochemistry, the spleen samples fixed with 4% (w/v) paraformaldehyde in PBS were cryoprotected in 30% (w/v) sucrose in PBS for 24 h at 4 °C followed by embedding in tissue freezing medium (Leica Biosystems) (71). The spleen sections of 7 μm thickness were then prepared using Leica CM1850 cryostat microtome and mounted on poly-L-lysine-coated slides. The sections were then blocked using 2% BSA for 2 h followed by incubation with Alexa Flour 488 anti-mouse IgD (Biolegend, 11-26c.2a) or biotin-conjugated anti-mouse GL7 (eBioscience Thermo Fisher, 13-5902-82) or Alexa Fluor 647 anti-mouse CD3 (Biolegend, 17A2) antibodies for 3 h at room temperature. The concentration of antibodies used was 2 μg/ml. For GL-7 detection, streptavidin-Alexa Flour 594 (Biolegend) was used at a concentration of 2.5 μg/ml. The sections were then washed, dried, and mounted with ProLong TM Gold antifade (Invitrogen). The fluorescence images were captured with a DP73 high-performance camera using an Olympus IX83 inverted microscope.

### Flow cytometry analyses

The spleen samples for the respective days were collected from the infected mice perfused with PBS. To isolate the splenocytes, the spleen samples were minced and digested with 2 mg/ml (Collagenase D > 0.15 U/mg, from Roche Diagnostics) in Collagenase D buffer containing 10 mM HEPES-NaOH, pH 7.4, 150 mM NaCl, 5 mM KCl, 1 mM MgCl_2_, and 1.8 mM CaCl_2_. The cells were then passed through a 70 μm nylon cell strainer followed by a 40 μm strainer to obtain the single-cell suspensions. The single-cell suspensions were then centrifuged at 300*g* for 10 min at 4 °C, and the pellets obtained were resuspended in 2 ml of RBC lysis buffer pH 7.4 containing 155 mM NH_4_Cl and 2 mM EDTA followed by 4 min incubation at room temperature. The cells were then washed and resuspended in PBS containing 0.5% BSA and 2 mM EDTA (70). For the flow cytometry analyses of cDCs and pDCs, DCs were enriched by subjecting the splenocytes to MACS cell separation using Pan Dendritic Cell Isolation Kit, mouse (Miltenyi Biotech, 130-100-875). The enriched DCs were then stained with anti-mouse CD11c-APC (Miltenyi Biotech, 130-102-800) and anti-mouse PDCA-1-FITC antibodies (Miltenyi Biotech, 130-102-827). For red pulp macrophage analysis, F4/80^+^ macrophages were first isolated using Anti-F4/80 Microbeads Ultrapure, mouse (Miltenyi Biotech, 130-110-443), followed by staining with anti-mouse CD11b-APC (Miltenyi Biotech; 130-113-802) and anti-mouse CD68-FITC (Biolegend; 137005). For the analyses of marginal zone and white pulp macrophages, splenocytes were stained with anti-mouse MHCII-PE (Miltenyi Biotech, 130-102-896), anti-mouse CD209b-Alexa Fluor 488 (eBioscience Thermo Fisher, eBIO22D1), anti-mouse CD169-APC (Biolegend, 142417) and anti-mouse CD68-FITC (Biolegend, 137005) antibodies. To analyse the levels of Tregs, CD4^+^CD25^+^ Tregs were isolated from the splenocytes using CD4^+^CD25^+^ Regulatory T Cell Isolation Kit, mouse (Miltenyi Biotech, 130-091-041). For Tfh cells, the flow-through fraction containing CD4^+^CD25^-^ cells obtained after the positive selection of CD4^+^CD25^+^ Tregs was stained with anti-mouse CXCR5-APC (Miltenyi Biotech, 130-119-129) and used for flow cytometry. For B cell analyses, B cells from the mouse spleen were isolated using B cell isolation kit (Miltenyi Biotech, 130-090-862) and stained with anti-mouse CD19-PE (Miltenyi Biotech, 130-111-883), anti-mouse CD21/CD35-PerCP-VIO700 (Miltenyi Biotech, 130-111-734), anti-mouse CD23-APC (Miltenyi Biotech,130-118-761), anti-mouse GL7-Biotin (eBioscience Thermo Fisher, 13-5902-82), anti-mouse IgD-VioBlue (Miltenyi Biotech, 130-102-268), anti-mouse IgM-PerCP-VIO700 (Miltenyi Biotech, 130-106-061) and anti-mouse IgG1-APC (Miltenyi Biotech, 130-102-308) antibodies. For the detection of markers stained with biotin-conjugated antibodies, Streptavidin-FITC (Miltenyi Biotech, 130-106-932) was used. All the antibodies were used at concentrations or dilutions recommended by the manufacturers. After incubating with the respective antibodies, cells were washed with PBS containing 0.5% BSA and 2 mM EDTA, and acquired using BD LSR Fortessa and CytoFLEX S. To compensate for the spectral overlap, single fluorochrome staining was performed for each experiment. The data were analyzed using FlowJo v10.6.1 software.

### Transcriptomic studies

*Pb* infections were carried out in Balb/c mice and the total RNA was extracted from *Pb*WT and *Pb*HMGB1KO parasites using RNeasy Mini Kit (Qiagen, 74104). The quality of RNA samples was assessed using Bioanalyzer (Agilent Technologies, Inc) and their concentrations were determined using Qubit (Thermo Fischer Scientific). For RNA-Seq library preparations, NEBNext Ultra II RNA Library Prep Kit for Illumina (NEB#E7770S) was utilized. Briefly, mRNAs isolated using magnetic oligo-d(T) beads were used for the first- and second-strand cDNA synthesis followed by the end preparation of the cDNA libraries and adapter ligations using the manufacturer’s protocols. The cDNA libraries were quantified using Qubit HS Assay (Invitrogen, Cat# Q32854) and the pooled libraries of 160 pM concentrations were then loaded on to 25B flow cells and sequenced using Illumina NovaSeq X Plus to generate 150 bp paired-end reads. The raw BCL files were converted to FASTQ files and the sequenced reads were demultiplexed. The quality checks were performed using the FastQC v.0.11.5 (http://www.bioinformatics.babraham.ac.uk/projects/fastqc/). *Pb*ANKA genome (v68) and its corresponding genomic feature files were downloaded from PlasmoDB. Genome index was generated in STAR (v2.5.3a) and the reads were mapped using the STAR RNA-Seq alignment tool. The count matrix for the mapped genes was generated using featureCounts v.1.6.4 from Subread package (74) and was used with Q = 10 for mapping quality, and these count files were used as input for downstream differential gene expression analysis with DESeq2 version 1.14.1. Normalization and differential expression were carried out using the DESeq2 Bioconductor package with the R statistical programming environment. The false discovery rate (FDR < 0.05) was calculated using Benjamini-Hochberg procedure to control multiple hypothesis testing. The genes exhibiting a substantial magnitude of fold-change (≥1.5 fold) with a stringent statistical threshold (adjusted *p*-value < 0.05) were classified as significantly differentially expressed. To generate the heatmaps, differential gene lists from each comparison were used along with their normalized values using complex heatmap Bioconductor package.

### Other procedures

Parasite isolations were carried out by lysing the infected RBC pellets with an equal volume of 0.15% saponin (Sigma-Aldrich, S4521) in PBS (w/v). The samples were then centrifuged at 10,000 g for 10 min at 4 °C to obtain the parasites and the supernatants collected after saponin lysis were used as iRBC lysates. The parasite lysates were prepared by sonicating the parasite pellets resuspended in lysis buffer containing 50 mM Tris pH 7.5, 100 mM NaCl, 0.05% Tween 20 (Sigma-Aldrich) and 1× Halt Protease Inhibitor Cocktail (Thermo Fisher Scientific). The lysates were then centrifuged at 30,000*g* for 15 min at 4 °C, and the supernatants were collected and used for further analyses. Protein estimations were carried out using a Micro BCA protein assay kit (ThermoFisher Scientific). To perform ELISA, wells were coated with the parasite or iRBC lysates or r*Pb*HMGB1 in 0.05 M carbonate buffer pH 9.6 and incubated overnight at 4 °C. Blocking was carried out with 3% BSA in PBS containing 0.1% Tween-20. Rabbit polyclonal GFP antibody (ab290, Abcam) and rabbit polyclonal serum raised against r*Pb*HMGB1 were used at 1:3000 dilution. HRP-conjugated goat anti-rabbit IgG secondary antibody (ab97051) was used 1:25,000 dilution. The HRP activity was detected using a tetramethylbenzidine liquid substrate and after stopping the reaction with 2 M H_2_SO_4_, the absorbance was measured at 450 nm. For the sensitivity assays, the percentage of blood parasitemia was calculated using Giemsa smears prepared from the tail vein blood, and the total number of RBCs present in the mouse blood collected for parasite preparations was estimated using a hemocytometer. Based on these, the total number of parasites present in the pellets obtained after saponin lysis was determined. The parasite lysates were prepared by sonication and centrifuged to remove membrane debris, and the lysates corresponding to the defined number of parasites were used to coat the wells of ELISA plates. *In vivo* bioluminescence studies were carried out as described (70, 71) using D-luciferin substrate (Promega VivoGlo). Immunization of rabbits to generate polyclonal sera against recombinant *Pb*HMGB1 was carried out using Freund’s complete and incomplete adjuvant (Sigma-Aldrich, F5881, and F5506). The specificity of HMGB1 antibodies was confirmed with HMGB1KO parasites. The splenectomy for mice was carried out as described earlier (75). SDS-PAGE and Western blot analyses were carried out following the standard protocols and the blots were developed using Pierce ECL Western Blotting Substrate (ThermoFisher Scientific, 32106). Genomic DNA isolation was carried out using a DNeasy Blood & Tissue Kit (Qiagen, 69506). Southern analysis was carried out using the standard protocol. DIG DNA Labeling Kit (Roche, 11093657910) was used to generate *Pb*HMGB1 3′UTR-specific probe labeled with Digoxigenin (DIG). The hybridization of the DIG-labelled probe was detected using DIG Luminescent Detection Kit (Roche, 11363514910).

### Statistical analyses

GraphPad Prism 8.0 (GraphPad Software) and Microsoft Excel were used to plot the graphs and analyze the data. Statistical analyses were carried out using two-way ANOVA, Tukey test, and two-tailed, unpaired *t* test. The log-rank (Mantel-Cox) test was used to determine the significance in survival curve analysis. Normalization and differential expression analysis of RNA-Seq were carried out using the DESeq2 Bioconductor package with the R statistical programming. The false discovery rate was calculated using Benjamini-Hochberg procedure to control multiple hypothesis testing.

## Data availability

All data are included within the article or supporting information. The raw files of RNA-Seq data are available at biostudies@ebi.ac.uk under accession number E-MTAB-14378.

## Supporting information

This article contains supporting information.

## Conflict of interest

The authors declare that they have no conflicts of interest with the contents of this article.
