# Supplementary material for: Plasmodium berghei HMGB1 controls the host immune responses and splenic clearance by regulating the expression of pir genes
Source: J Biol Chem. 2024 Sep 27;300(11):107829. doi: 10.1016/j.jbc.2024.107829 (PMC11541847; doi:10.1016/j.jbc.2024.107829)
Supplement: Supporting information [file mmc1.pdf]

SUPPORTING INFORMATION

Plasmodium berghei HMGB1 controls the host immune responses and splenic clearance by regulating the expression of *pir* genes

Pradeep Mini Vaishalli[1,2,6], Rahul Das[1,2,6], Harveer Singh Cheema[1,3,6], Sourav Ghosh[1,2], Manjunatha Chandana[1], Aditya Anand[1,2], Krushna Chandra Murmu[4], Govindarajan Padmanaban[5], Balachandran Ravindran[1] and Viswanathan Arun Nagaraj[1,*]

[1]Infectious Disease Biology, Institute of Life Sciences, Bhubaneswar - 751023, Odisha, India

[2]Regional Centre for Biotechnology, Faridabad - 121001, Haryana, India

[3]Department of Botany, Meerut College, Meerut - 250003, Uttar Pradesh, India

[4]PharmaFace, Begumpet, Hyderabad- 500016, Telangana, India

[5]Department of Biochemistry, Indian Institute of Science, Bangalore - 560012, Karnataka, India

[6]These authors contributed equally

*Corresponding author: Email: arun@ils.res.in

Running Title: The gene regulatory function of *Pb*HMGB1

Supplementary Figures 1-10

Supplementary Table 1

Description of Supplementary Dataset 1

Supplementary Figures

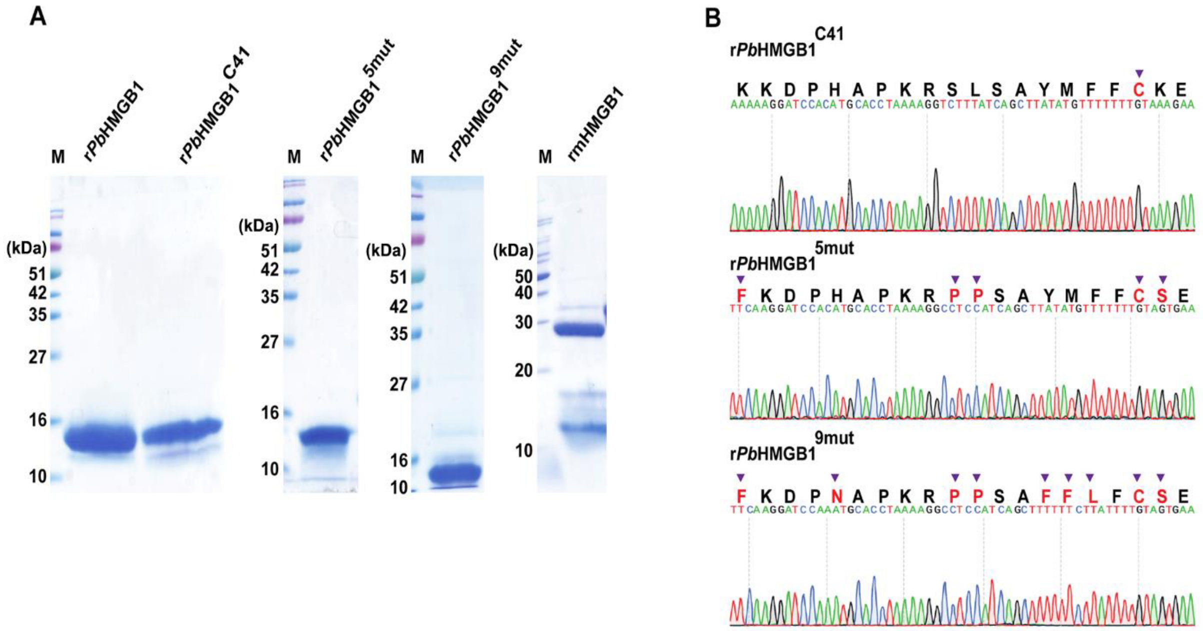

Figure S1: Site-directed mutagenesis of r*Pb*HMGB1. (**A**) Coomassie stained images of purified r*Pb*HMGB1, r*Pb*HMGB1^{5mut}, r*Pb*HMGB1^{9mut} and rmHMGB1 resolved in SDS-PAGE. Lane M: Protein molecular weight marker (kDa). (**B**) Chromatograms of DNA sequencing performed for the plasmids of r*Pb*HMGB1, r*Pb*HMGB1^{5mut} and r*Pb*HMGB1^{9mut}. The respective mutations are indicated with purple arrow heads.

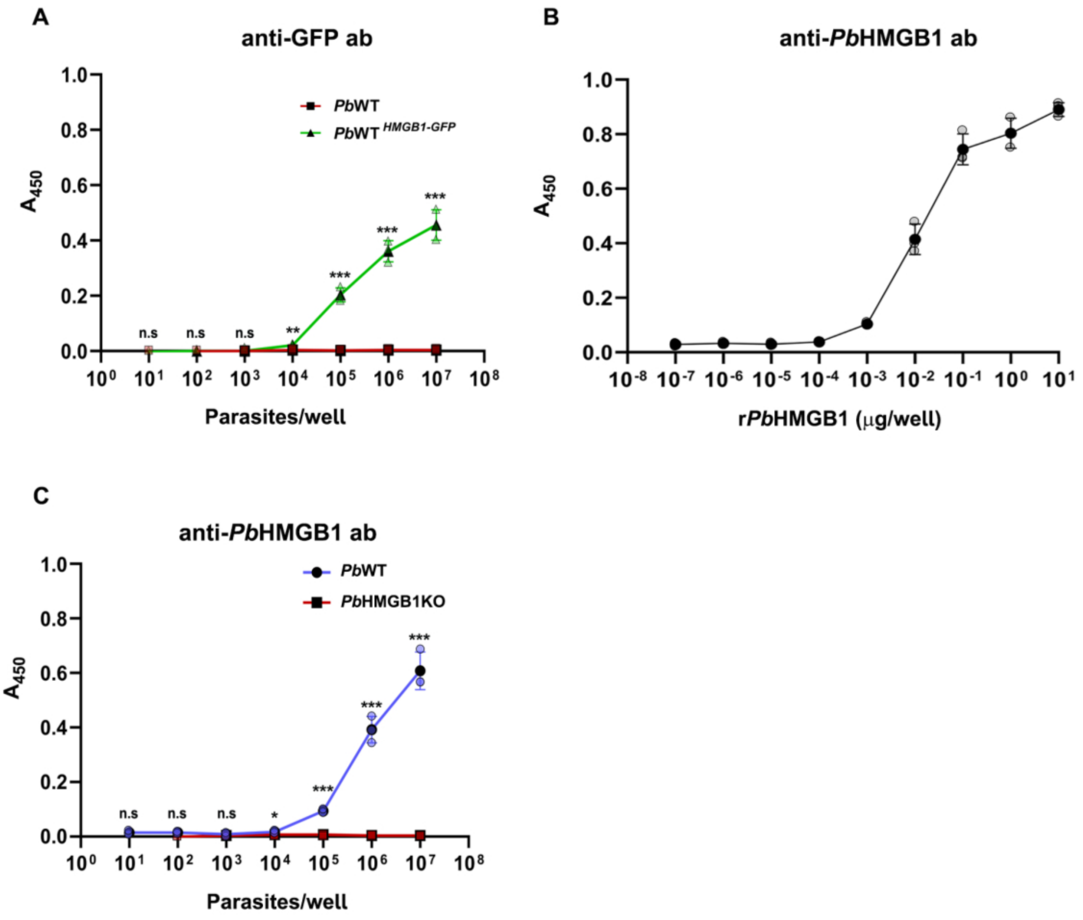

Figure S2: Sensitivity of anti-GFP antibodies and anti-*Pb*HMGB1 polyclonal sera. (**A**) ELISA assays performed with anti-GFP antibodies for the lysates representing the defined number of *Pb*WT$^{HMGB1\text{-}GFP}$ and *Pb*WT parasites. (**B**) ELISA assays performed with anti-*Pb*HMGB1 polyclonal sera for r*Pb*HMGB1. (**C**) ELISA assays performed with anti-*Pb*HMGB1 polyclonal sera for the lysates representing the defined number of *Pb*WT and *Pb*HMGB1KO parasites. The data (mean ± SD) represent three independent preparations (n.s. - not significant, **P*<0.05, ***P*<0.01, ****P*<0.001; unpaired t-test; two-tailed).

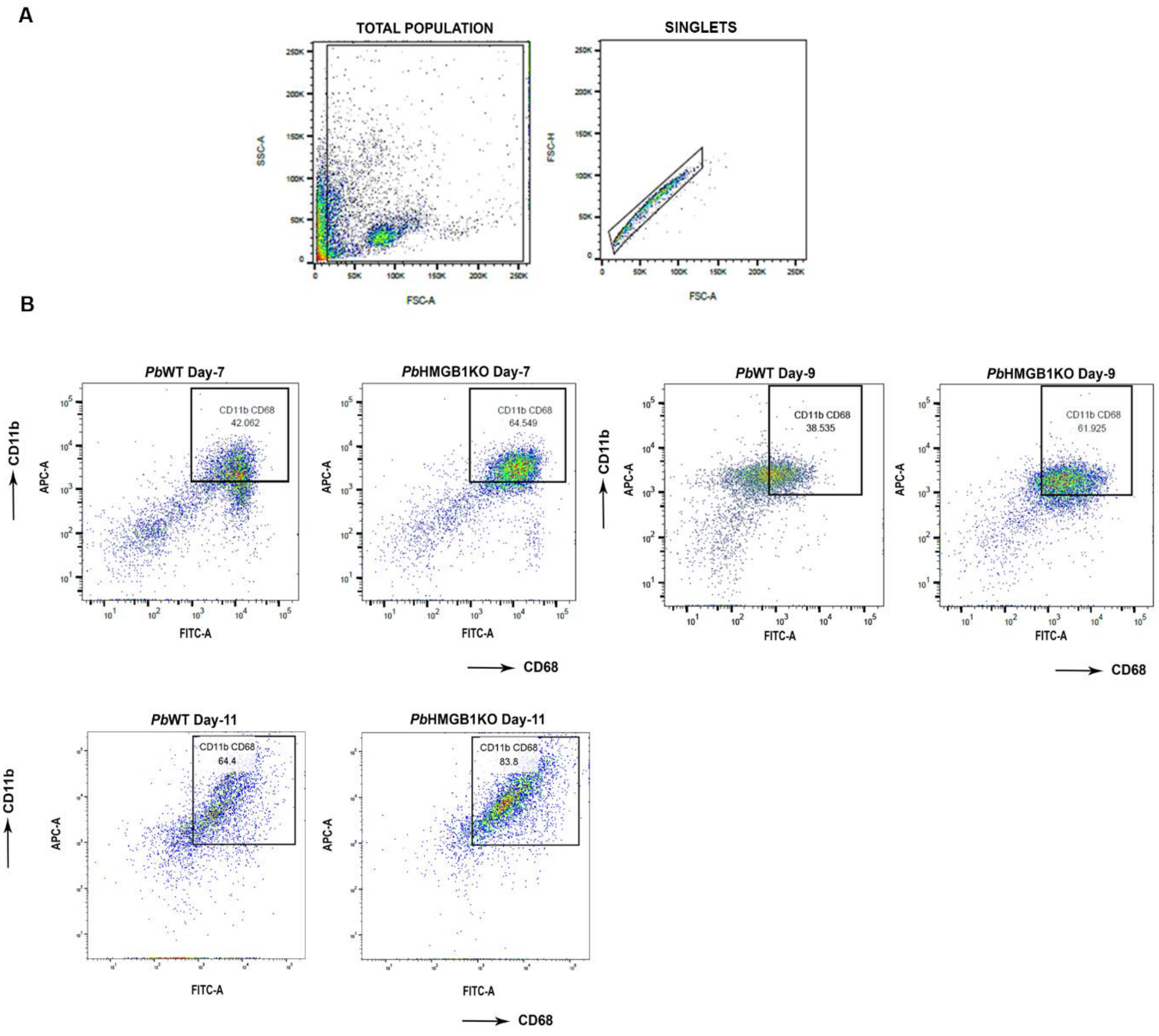

Figure S3: Flow cytometry analysis of red pulp macrophages. (**A**) Gating strategy. (**B**) Representative plots for *Pb*WT- and *Pb*HMGB1KO-infected mouse spleen samples are provided.

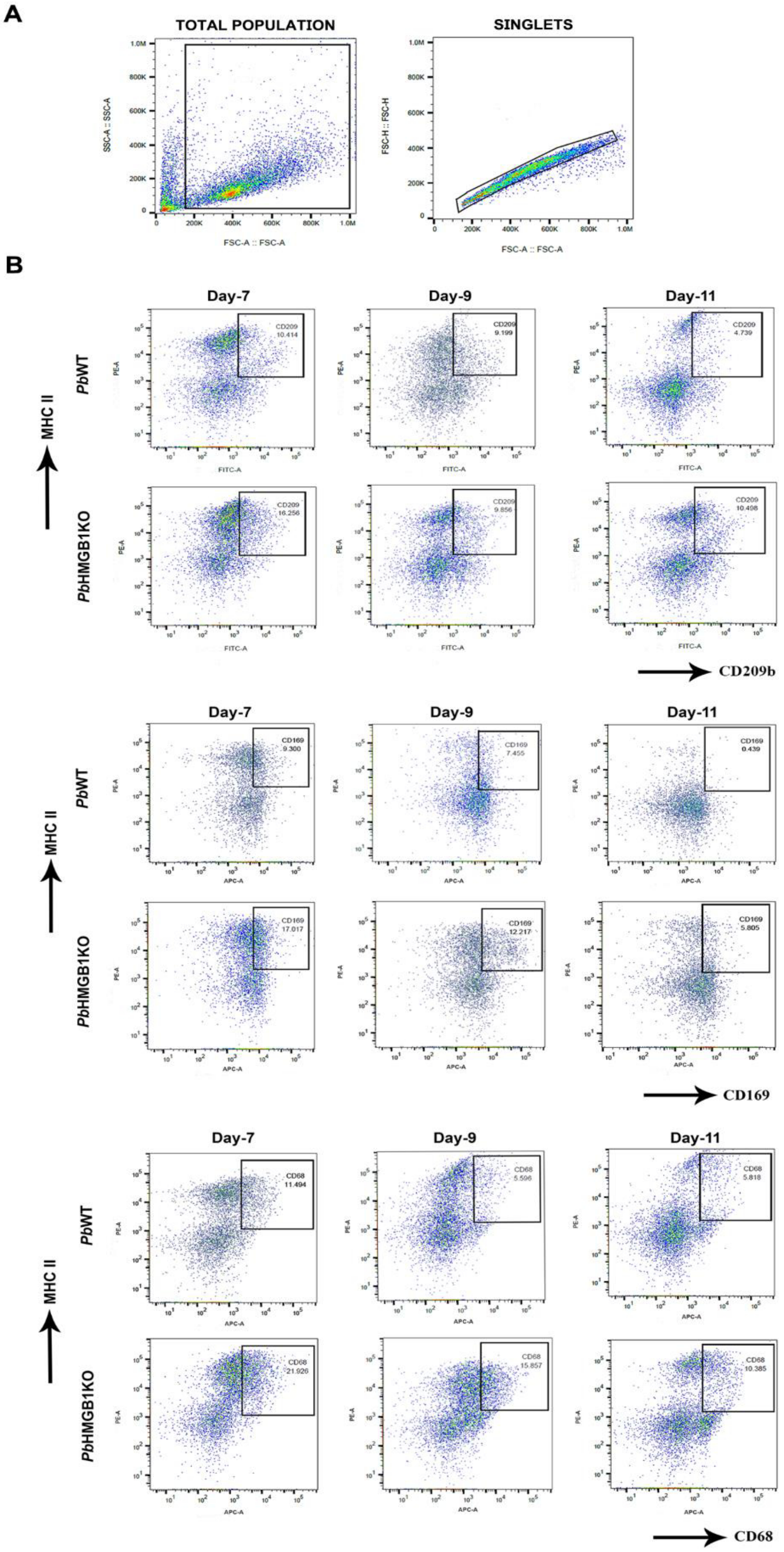

Figure S4: Flow cytometry analysis of marginal zone and white pulp macrophages. (**A**) Gating strategy. (**B**) Representative plots for *Pb*WT- and *Pb*HMGB1KO-infected mouse spleen samples are provided.

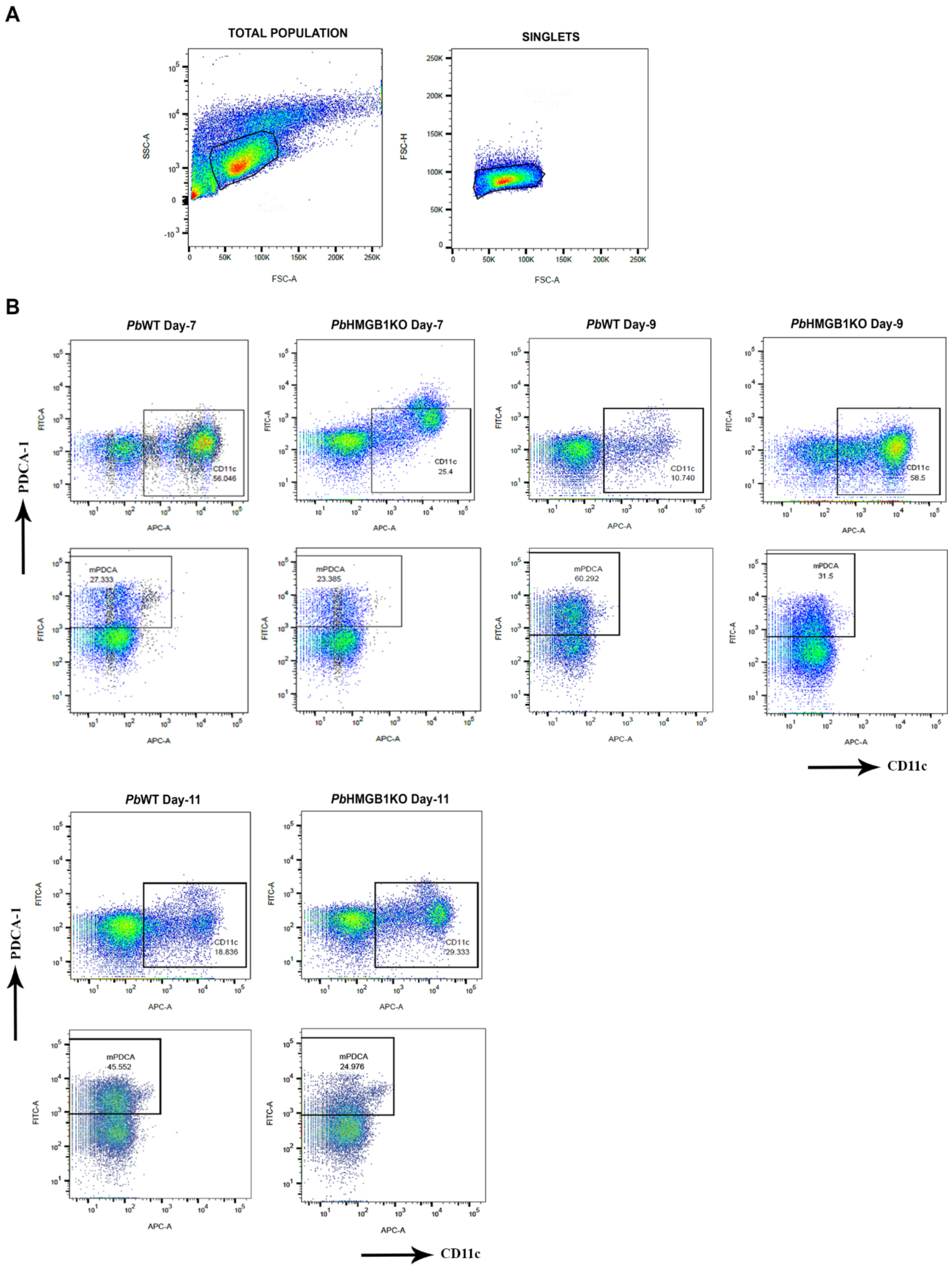

Figure S5: Flow cytometry analysis of conventional and plasmacytoid dendritic cells. (**A**) Gating strategy. (**B**) Representative plots for *Pb*WT- and *Pb*HMGB1KO-infected mouse spleen samples are provided.

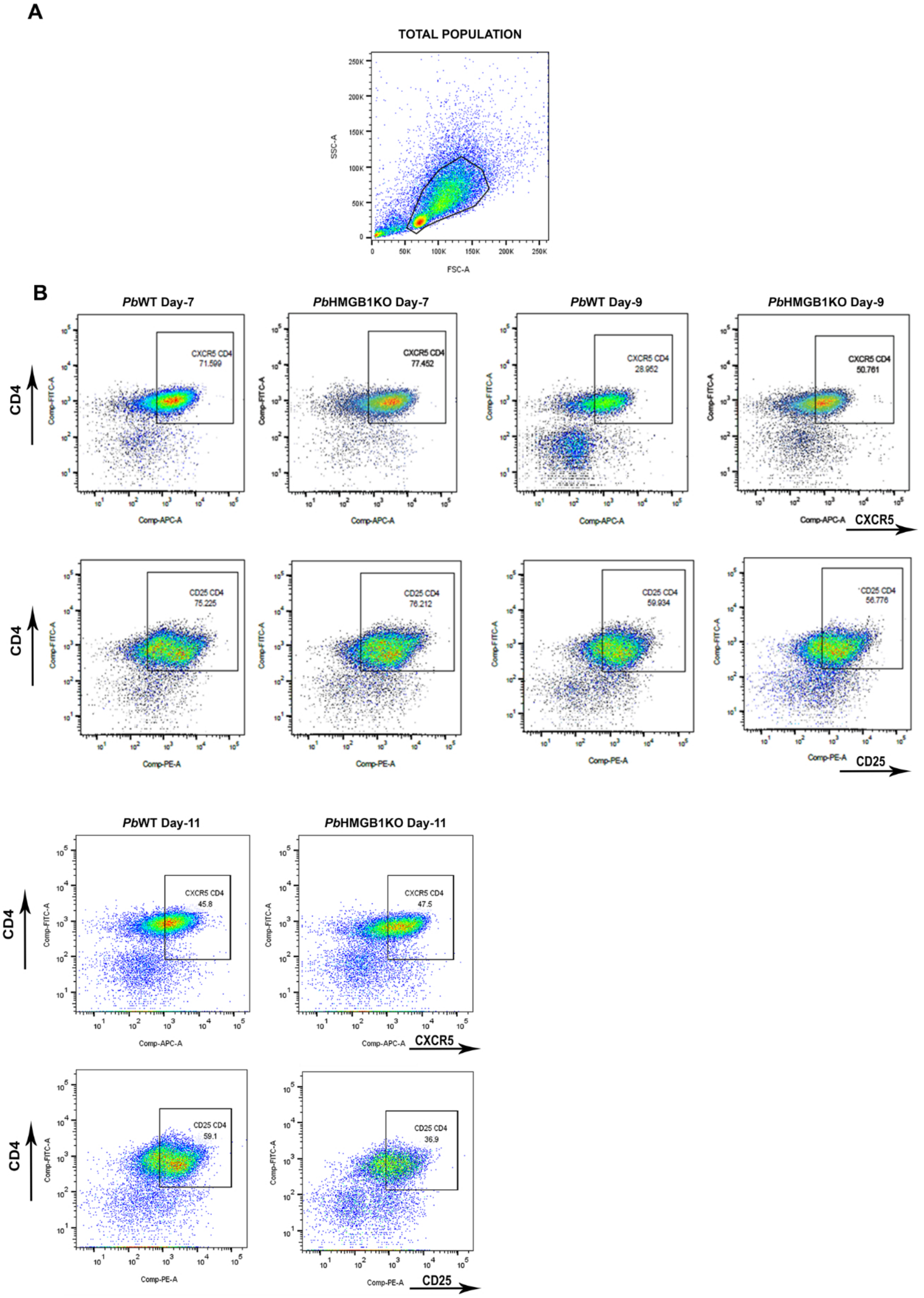

Figure S6: Flow cytometry analysis of T-follicular helper cells and regulatory T cells. (**A**) Gating strategy. (**B**) Representative plots for *Pb*WT- and *Pb*HMGB1KO-infected mouse spleen samples are provided.

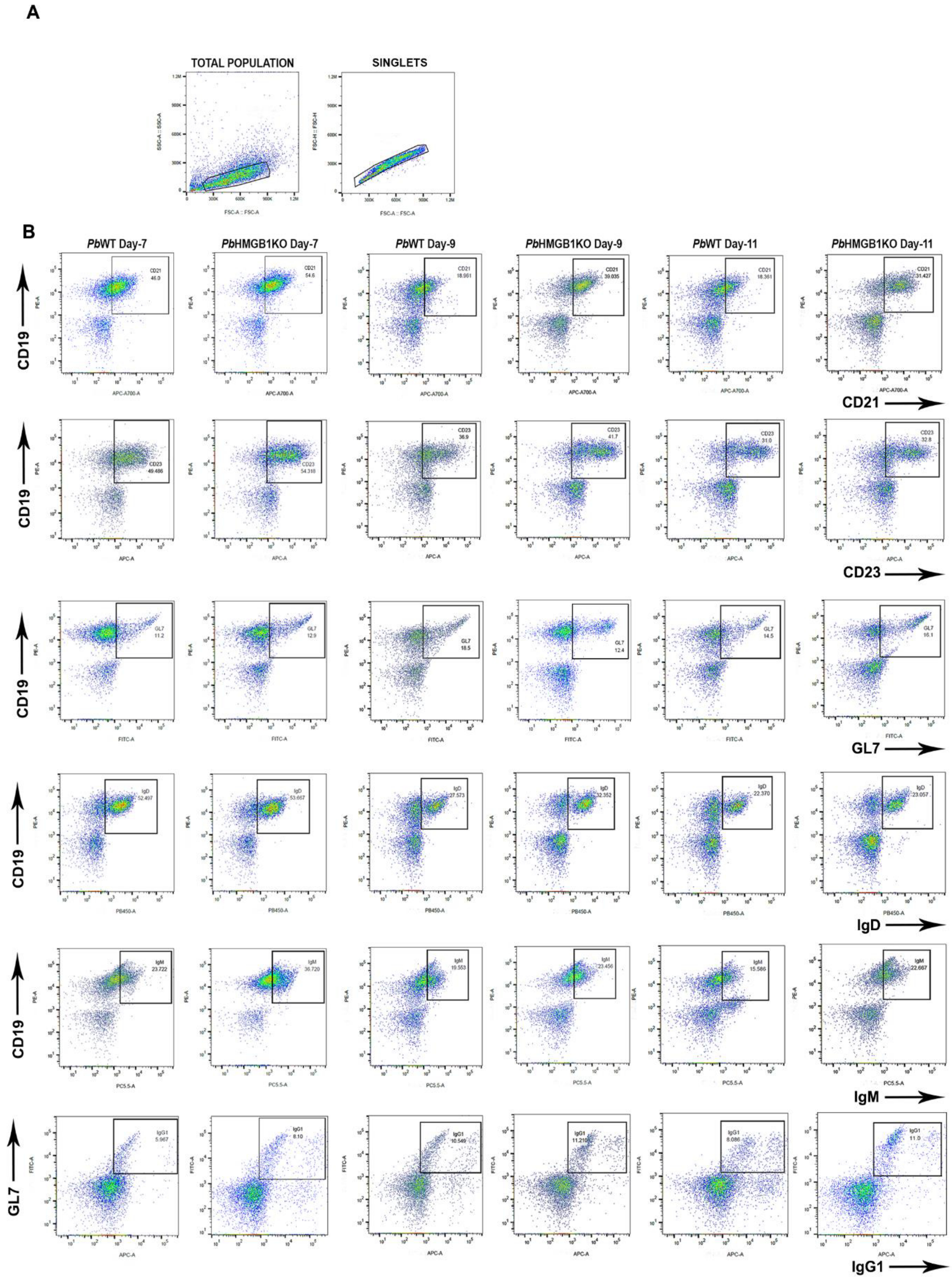

Figure S7: Flow cytometry analysis of marginal zone, follicular and germinal center B cells. (**A**) Gating strategy. (**B**) Representative plots for *Pb*WT- and *Pb*HMGB1KO-infected mouse spleen samples are provided.

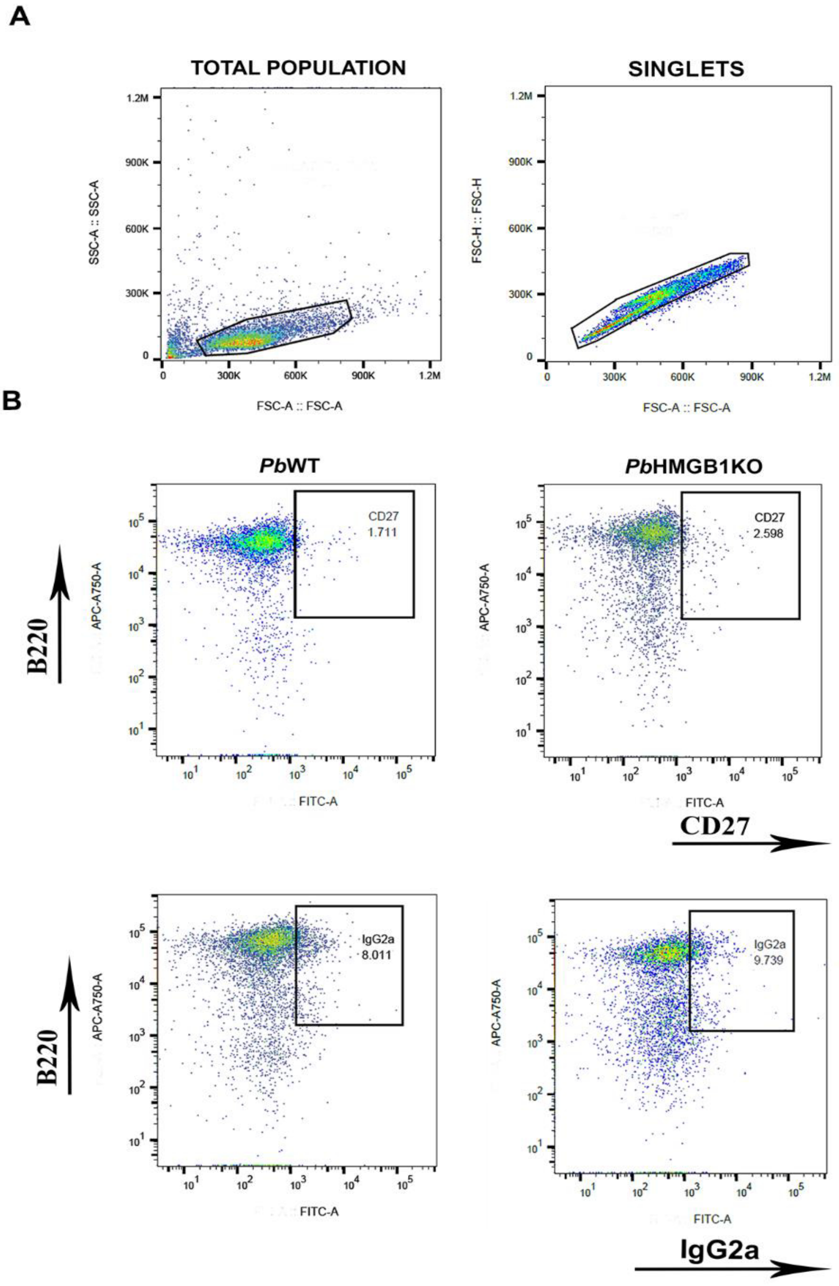

Figure S8: Flow cytometry analysis of memory B cells. (**A**) Gating strategy. (**B**) Representative plots for *Pb*WT- and *Pb*HMGB1KO-infected mouse spleen samples are provided.

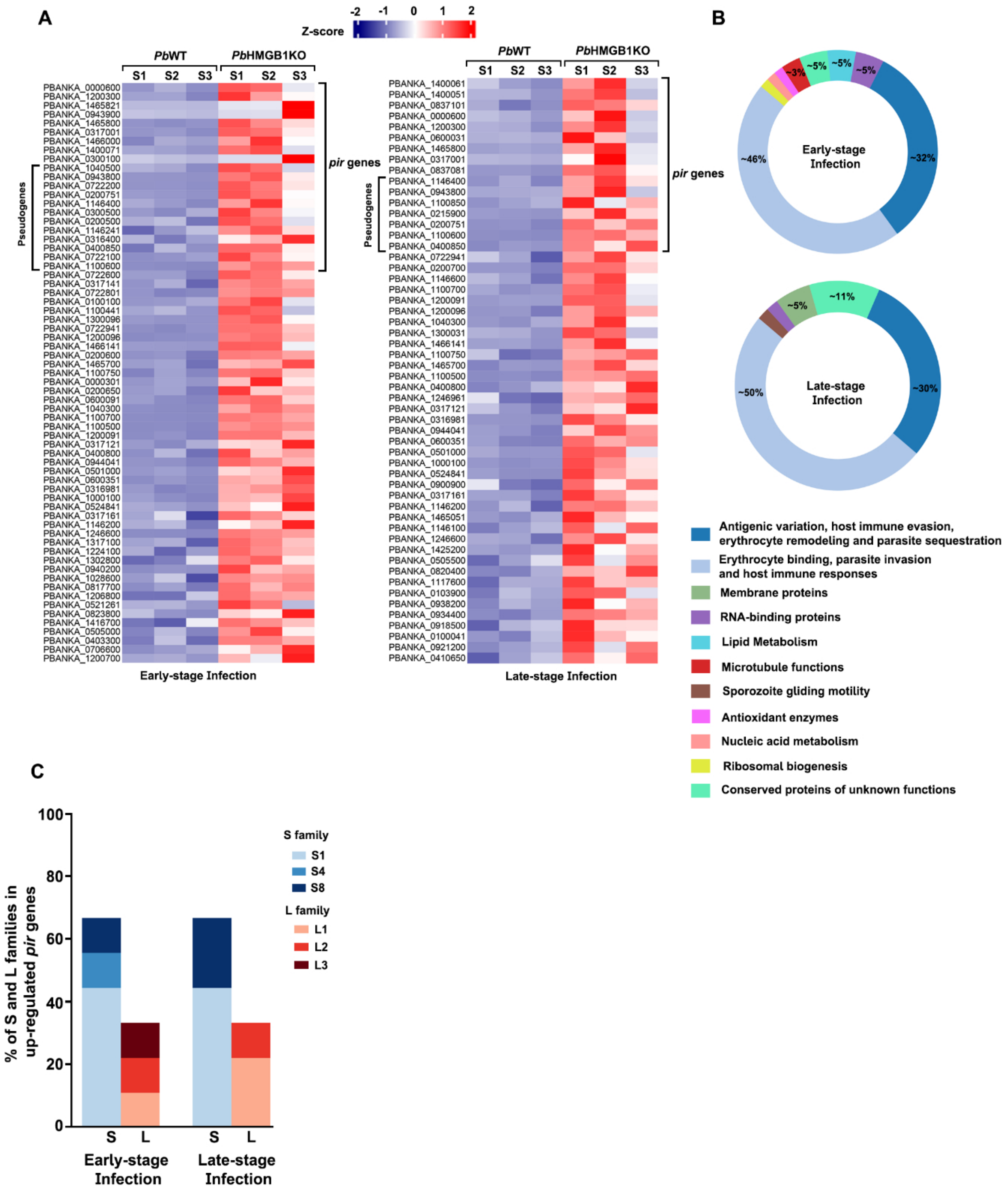

Figure S9: Transcriptomics of *Pb*HMGB1KO parasites. (A) List of up-regulated genes in *Pb*HMGB1KO parasites during early- and late-stage infections. The genes that showed significant up-regulation with greater than 1.5-fold change, FDR < 0.05 and adjusted p-value < 0.05 were considered (Benjamini-Hochberg procedure; multiple hypothesis testing). The data represent three independent parasite pellets of *Pb*WT and *Pb*HMGB1KO parasites for early- and late-stage infections, respectively. The down-regulated genes are provided in Figure 10.

(**B**) Donut chart representing the gene ontologies of significantly up-regulated genes based on the functional annotations available in PlasmoDB and published literature. (**C**) Percentage of S and L families in the up-regulated *pir* genes and the proportion of various clades. The entire details of RNA-Seq analyses are provided in Supplementary Dataset 1.

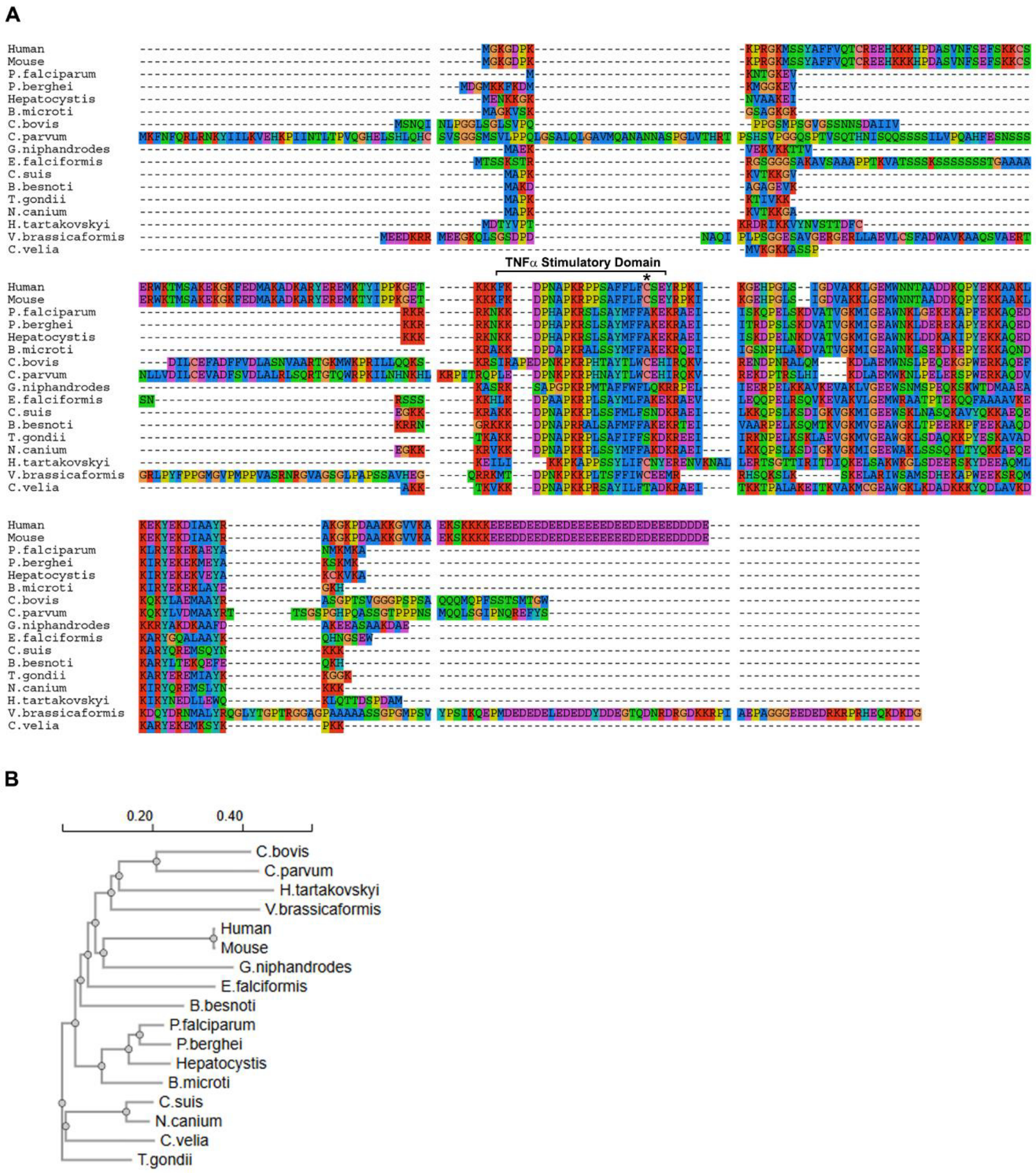

Figure S10: Sequence comparison of alveolate HMGBs. (**A**) Multiple protein sequence alignment showing the sequence homology of TNF-α stimulatory domain of mouse and human HMGB1 with B box of alveolate HMGBs. Cys106 present in the TNF-α stimulatory domain of mammalian (mouse and human) HMGB1 is conserved in *Vitrellla brassicaformis*, *Haemoproteus tartakovskyi*, *Cryptosporidium bovis* and *Cryptosporidium parvum* HMGBs. HMGB sequences of *Hepatocystis* (HEP_00168200), *Babesia microti* (BMR1_01G01876), *C. bovis* (FG379_002132), *C. parvum* (cgd8_4220), *G. niphandrodes* (GNI_091770), *Eimeria falciformis* (EfaB_MINUS_15648.g1375), *Cystoisospora suis* CSUI_010949, *Besnoitia besnoti* (BESB_000410), *Toxoplasma gondii* (TGME49_210408), *Neospora canium*

(NCLIV_060790), *H. tartakovskyi* (Htart_000218300), *V. brassicaformis* (Vbra_19211) and *Chromera velia* (Cvel_2419) were retrieved from PlasmoDB (https://plasmodb.org/plasmo/app), ToxoDB (https://toxodb.org/toxo/app/), CryptoDB (https://cryptodb.org/cryptodb/app), and PiroplasmaDB (https://piroplasmadb.org/piro/app). Cys 106 of mammalian HMGB1 is highlighted with asterisk. (**B**) The respective phylogram of the sequence alignment. Multiple protein sequence alignment was carried out with SeaView Version 5.0.5 (https://doua.prabi.fr/software/seaview).

Supplementary Table 1: Primers used for site-directed mutagenesis to generate r*Pb*HMGB1^{C41}, r*Pb*HMGB1^{5mut} and r*Pb*HMGB1^{9mut}. The order of mutation represents the sequence followed for the generation of these mutants. The mutated nucleotides in the primers are underlined. The mutant plasmid generated for the previous mutation was used as a template for the next mutation.

**Order of mutation**	**Position of mutation**	**Primers used for mutagenesis (5'-3')**
1st mutation (r*Pb*HMGB1^{C41})	A41C	CAGCTTATATGTTTTTT**TGT**AAAGAAAAGAGAGCAG<br>CTGCTCTCTTTTCTTT**ACA**AAAAAACATATAAGCTG
2nd mutation	K42S	CAGCTTATATGTTTTTTTGT**AGT**GAAAAGAGAGCAG<br>CTGCTCTCTTTTCAC**TAC**AAAAAAACATATAAGCTG
3rd mutation	K24F	GAAACGAAGAAAAAAT**TTC**AAGGATCCACATGCACCT<br>AGGTGCATGTGGATCCTT**GAA**ATTTTTCTTCGTTTC
4th mutation	L34P	CATGCACCTAAAAGGTCT**CCA**TCAGCTTATATGTTTTTTG<br>CAAAAAACATATAAGCTGA**TGG**AGACCTTTTAGGTGCATG
5th mutation (r*Pb*HMGB1^{5mut})	S33P	CCACATGCACCTAAAAGG**CCT**CCATCAGCTTATATGTTTTTTGTAG<br>CTACAAAAAAACATATAAGCTGA**TGG**AGGCCTTTTAGGTGCATGTGG
6th mutation	H28N	GAAAAAATTTCAAGGATCCA**AAT**GCACCTAAAAGGCCTCC<br>GGAGGCCTTTTAGGTGC**ATT**TGGATCCTTGAAATTTTTC
7th mutation	Y37F	GGCCTCCATCAGC**TTT**TATGTTTTTTGTAG<br>CTACAAAAAAACAT**AAA**AGCTGATGGAGGCC
8th mutation	M38F	CCTCCATCAGCTTTT**TTC**TTTTTTGTAGTG<br>CACTACAAAAAAA**GAA**AAAAGCTGATGGAGG

9th mutation<br><br>(r*Pb*HMGB1 9mut)	F39L	CCATCAGCTTTTTTC**TTA**TTTTGTAGTGAAAAG<br><br>CTTTTCACTACAAAA**TAA**GAAAAAAGCTGATGG

Description of Supplementary Dataset

Supplementary Dataset 1: Details of RNA-Seq analyses. Normalized counts of all the transcripts in the early- (ES) and late-stage (LS) infections of *Pb*WT and *Pb*HMGB1KO parasites. The list of genes that showed significant down-regulation and up-regulation in the early- and late-stage infections of *Pb*HMGB1KO parasites along with their functional annotations. The families and clades of *pir* genes are also provided.
